# Seasonal and Geographical Transitions in Eukaryotic Phytoplankton Community Structure in the Atlantic and Pacific Oceans

**DOI:** 10.3389/fmicb.2020.542372

**Published:** 2020-09-30

**Authors:** Chang Jae Choi, Valeria Jimenez, David M. Needham, Camille Poirier, Charles Bachy, Harriet Alexander, Susanne Wilken, Francisco P. Chavez, Sebastian Sudek, Stephen J. Giovannoni, Alexandra Z. Worden

**Affiliations:** ^1^Ocean EcoSystems Biology Unit, GEOMAR Helmholtz Centre for Ocean Research Kiel, Kiel, Germany; ^2^Monterey Bay Aquarium Research Institute, Moss Landing, CA, United States; ^3^Biology Department, Woods Hole Oceanographic Institution, Woods Hole, MA, United States; ^4^Institute for Biodiversity and Ecosystem Dynamics, University of Amsterdam, Amsterdam, Netherlands; ^5^Department of Microbiology, Oregon State University, Corvallis, OR, United States

**Keywords:** dictyochophytes, phytoplankton diversity, time-series, single-cell genomics, chloroplast genome

## Abstract

Much is known about how broad eukaryotic phytoplankton groups vary according to nutrient availability in marine ecosystems. However, genus- and species-level dynamics are generally unknown, although important given that adaptation and acclimation processes differentiate at these levels. We examined phytoplankton communities across seasonal cycles in the North Atlantic (BATS) and under different trophic conditions in the eastern North Pacific (ENP), using phylogenetic classification of plastid-encoded 16S rRNA amplicon sequence variants (ASVs) and other methodologies, including flow cytometric cell sorting. Prasinophytes dominated eukaryotic phytoplankton amplicons during the nutrient-rich deep-mixing winter period at BATS. During stratification (‘summer’) uncultured dictyochophytes formed ∼35 ± 10% of all surface plastid amplicons and dominated those from stramenopile algae, whereas diatoms showed only minor, ephemeral contributions over the entire year. Uncultured dictyochophytes also comprised a major fraction of plastid amplicons in the oligotrophic ENP. Phylogenetic reconstructions of near-full length 16S rRNA sequences established 11 uncultured Dictyochophyte Environmental Clades (DEC). DEC-I and DEC-VI dominated surface dictyochophytes under stratification at BATS and in the ENP, and DEC-IV was also important in the latter. Additionally, although less common at BATS, *Florenciella*-related clades (FC) were prominent at depth in the ENP. In both ecosystems, pelagophytes contributed notably at depth, with PEC-VIII (Pelagophyte Environmental Clade) and (cultured) *Pelagomonas calceolata* being most important. Q-PCR confirmed the near absence of *P. calceolata* at the surface of the same oligotrophic sites where it reached ∼1,500 18S rRNA gene copies ml^–1^ at the DCM. To further characterize phytoplankton present in our samples, we performed staining and at-sea single-cell sorting experiments. Sequencing results from these indicated several uncultured dictyochophyte clades are comprised of predatory mixotrophs. From an evolutionary perspective, these cells showed both conserved and unique features in the chloroplast genome. In ENP metatranscriptomes we observed high expression of multiple chloroplast genes as well as expression of a selfish element (group II intron) in the *psaA* gene. Comparative analyses across the Pacific and Atlantic sites support the conclusion that predatory dictyochophytes thrive under low nutrient conditions. The observations that several uncultured dictyochophyte lineages are seemingly capable of photosynthesis and predation, raises questions about potential shifts in phytoplankton trophic roles associated with seasonality and long-term ocean change.

## Introduction

Open ocean ecosystems undergo seasonal changes that influence water column vertical structure and productivity, and these ecosystems are predicted to expand under future ocean conditions (Flombaum et al., 2013; Worden et al., 2015). The Bermuda Atlantic Time-series Study (BATS) is located in a seasonally oligotrophic subtropical gyre in the Sargasso Sea (Carlson et al., 1994; Steinberg et al., 2001; Lomas et al., 2013). Pronounced seasonality at BATS is reflected in winter deep-mixing and strong summer thermal stratification, making it an exceptional site for studying transitions in plankton communities associated with warming oceans and declining productivity (Carlson et al., 1994; Steinberg et al., 2001; Lomas et al., 2013). In average years at BATS, deep vertical mixing entrains nutrients into the photic zone during the winter months, supporting relatively high primary productivity in spring, followed by strong thermal stratification and lower summertime productivity. The seasonal dynamics of phytoplankton communities track these periods, transitioning from eukaryotic phytoplankton dominance in the winter and spring, to cyanobacterial dominance in summer and fall, based on high-performance liquid chromatography (HPLC) analysis of pigments and numerical abundance by flow cytometry (Durand et al., 2001; Lomas et al., 2010). Further, although eukaryotic phytoplankton number less than cyanobacteria in this subtropical gyre, they often comprise a significant proportion of phytoplankton biomass (Goericke, 1998; Durand et al., 2001; Cuvelier et al., 2010; Lomas et al., 2010).

While cyanobacterial communities have been well studied at BATS (Malmstrom et al., 2010), characterization of eukaryotic phytoplankton here and at other oligotrophic ocean settings has mainly focused on broad taxonomic categories. At BATS, an early feature of the winter/spring cycle of phytoplankton turnover is a prasinophyte bloom, based on terminal restriction fragment length polymorphism (T-RFLP) analysis and limited qPCR data (Treusch et al., 2012), while afterwards haptophytes and pelagophytes appear to rise in importance based on HPLC pigment analyses and limited molecular data (Goericke, 1998; Cuvelier et al., 2010). Apart from the haptophytes (Cuvelier et al., 2010), the specific taxa that comprise eukaryotic phytoplankton communities at BATS have not been characterized using high throughput molecular marker gene sequencing approaches that help to identify patterns at finer taxonomic scales.

Global scale efforts like TARA Oceans (de Vargas et al., 2015) have found a diversity of stramenopiles across the oceans. Stramenopiles include algae that are considered important in higher latitude and coastal systems, such as diatoms, which appear to bloom only rarely at BATS (Steinberg et al., 2001; Lomas et al., 2010). Apart from diatoms, other relatively well studied stramenopile algal classes include pelagophytes (Andersen et al., 1993, 1996) and chrysophytes (Hibberd, 1976; Andersen et al., 1999), and lesser known ones include the bolidophytes (Guillou et al., 1999) and dictyochophytes (Henriksen et al., 1993; Andersen, 2004). The ecology of the former two lineages has been studied with multiple methods in various ocean regions, including BATS. These studies utilized approaches such as HPLC and T-RFLP (Andersen et al., 1996; Goericke, 1998; Steinberg et al., 2001; Treusch et al., 2012). However, one serious issue is that some stramenopile classes, like the dictyochophytes, cannot be discriminated from diatoms and pelagophytes using HPLC (Guillou et al., 1999; Daugbjerg and Henriksen, 2001). Further, the paucity of sequence data from a broad range of characterized stramenopiles or taxonomically verified environmental sequences has restricted studies of diversity and distributions. Hence, although distributions of pelagophytes (and some other stramenopiles) have been described at BATS based on HPLC data (Steinberg et al., 2001), the seasonal patterns and depth distributions for specific pelagophyte taxa are as yet unknown. In the Mediterranean Sea and South Pacific Ocean, analyses of the plastid-derived *psbA* gene and rRNA genes have indicated the presence of uncultured pelagophytes (Man-Aharonovich et al., 2010; Shi et al., 2011), while targeted metagenomic approaches connected to traditional metagenomic analyses indicate that pelagophytes highly similar to *Pelagomonas calceolata* are distributed across subtropical surface ocean waters (Worden et al., 2012). Further, in the Pacific Ocean this species can dominate 0.1-20 μm size fractionated samples from the subsurface chlorophyll maximum (SCM), based on metagenomics analyses in coastal California (Dupont et al., 2015).

Perhaps one of the most enigmatic stramenopile algal classes is the dictyochophytes. They have few cultured representatives, little biogeographical data apart from TARA (de Vargas et al., 2015), and little seasonal data. This group contains the silicoflagellates, which can be microscopically identified by their distinctive siliceous skeletons, and have been studied mainly in coastal northern ecosystems and sediments or as paleoecological markers (Henriksen et al., 1993; Rigual-Hernandez et al., 2016; van de Poll et al., 2018). Indeed, there are few oceanic time-series analyses that tease apart the contributions of diatoms, chrysophytes, and pelagophytes, and more newly discovered groups like bolidophytes (Goericke, 1998; Steinberg et al., 2001; Lomas et al., 2013), or those with few cultured representatives, like the dictyochophytes. Perhaps the best time-series molecular characterization has been performed at the San Pedro Ocean Time-series (SPOT) in the eastern North Pacific (ENP) off coastal USA where several dictyochophyte Amplicon Sequence Variants (ASVs) were reported as being common (Needham et al., 2018). The ENP differs dramatically from BATS, in part due to the paucity of phosphate in the latter as compared to the former (Coleman and Chisholm, 2010). In addition to SPOT, eukaryotic phytoplankton dynamics have been extensively studied in the ENP using classical approaches such as HPLC and microscopy as part of the California Cooperative Oceanic Fisheries Investigation (CalCOFI), which samples multiple transect ‘lines’ that run perpendicular to the California, United States coast on an approximately bimonthly basis (Collins et al., 2003; McClatchie, 2014). These transect lines typically cross the coastal zone, traverse productive upwelling waters and continue outward to the edge of the North Pacific Subtropical Gyre. Along the ENP’s CalCOFI Line-67, which extends from Monterey Bay to 800 km offshore, cyanobacterial diversity has been relatively well described at the level of molecular diversity (Sudek et al., 2015), and quantitative data exists for some algal groups, based on methods such as qPCR (Simmons et al., 2016; Kolody et al., 2019), HPLC and microscopy (Paerl et al., 2011; Chavez et al., 2017). However, less is known about the general molecular diversity of eukaryotic algae in the different trophic regimes of the ENP.

Here, we examined eukaryotic phytoplankton community structure in the Sargasso Sea over seasonal cycles and compared this data to algal distributions along the ENP Line-67. V1-V2 16S rRNA gene amplicon data from BATS photic-zone profiles (Treusch et al., 2009) were analyzed using phylogenetic approaches (Matsen et al., 2010; Choi et al., 2017). At BATS, we found a dominance of non-diatom stramenopile-derived amplicons in the multi-year dataset. This prompted us to develop well-curated, full-length 16S reference alignments and phylogenetic reconstructions for the most highly represented stramenopile groups, specifically the pelagophytes and dictyochophytes. The reconstructions provided new insights into clade diversity within these groups, as well as spatiotemporal patterns of fine-scale phytoplankton diversity. Comparison to community structure at Line-67 mesotrophic and oligotrophic sites highlighted similarities in phytoplankton community composition, with parallels between the influences of the seasonal cycle of nutrient availability at BATS and variation in nutrients along Line-67. To evaluate amplicon-based relative abundance patterns, a qPCR primer probe set was developed and implemented to enumerate *Pelagomonas calceolata*, which was identified as being the most abundant pelagophyte in amplicon data. Finally, because a prior Pacific Ocean 18S rRNA stable isotope probing experiment indicated that bolidophytes and a *Florenciella*-like dictyochophyte may act as mixotrophic predators (Frias-Lopez et al., 2009), we also performed single-cell food-vacuole-staining / chlorophyll-based cell sorting (Wilken et al., 2019) in order to capture potential mixotrophs among the wild phytoplankton taxa that were abundant in our flow cytometric histograms. This led to sequencing of uncultured dictyochophytes that appear to be predatory mixotrophs (present in the field sort experiments) and rendered the complete chloroplast genome of one such species. Collectively, the similarities observed between the two ecosystems, BATS and the ENP, were found to be especially strong for predatory mixotrophic stramenopiles in surface oligotrophic environments, based on trends that emerged when sequence data was parsed at high taxonomic resolution and with the aid of single-cell sort data from wild dictyochophytes.

## Results

### Eukaryotic Phytoplankton in the Western North Atlantic

**FIGURE 1:**
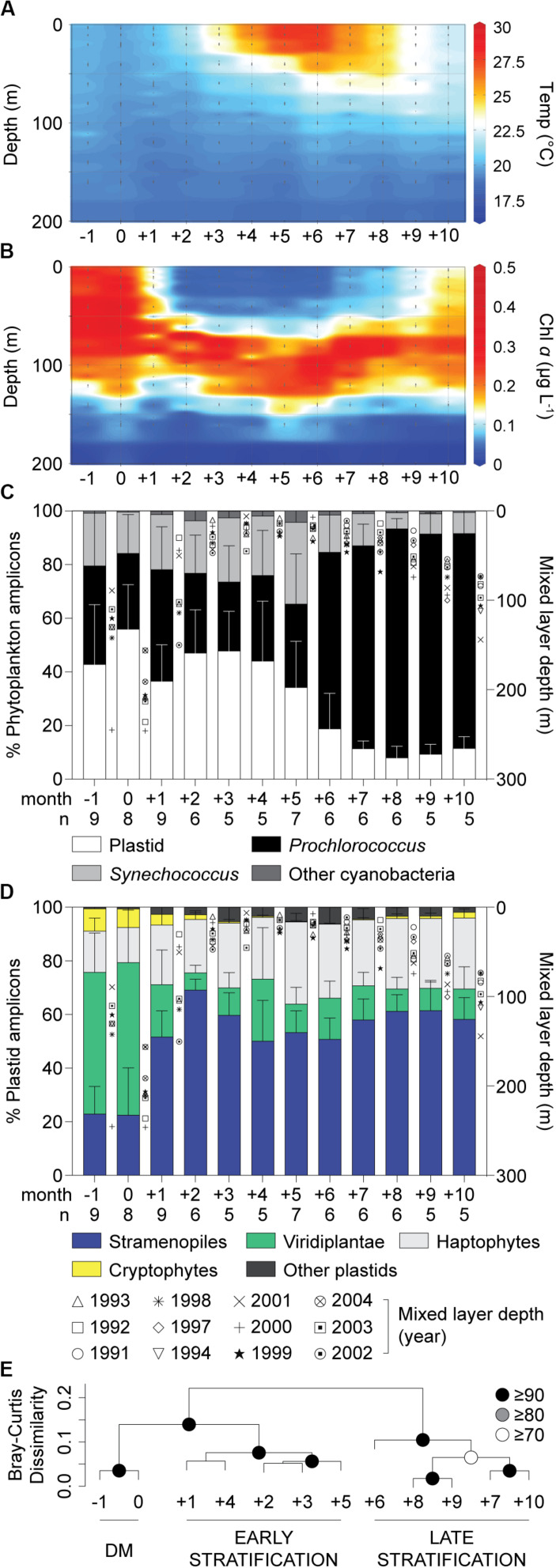
Characteristics of the water column and phytoplankton distributions in the northwestern Sargasso Sea. **(A)** Mean temperature and **(B)** chlorophyll *a* concentrations show the oceanographic details in this study site. **(C)** The relative abundance of overall phytoplankton 16S V1-V2 amplicons expressed as a percentage of amplicons phylogenetically assigned to plastids, *Prochlorococcus*, *Synechococcus* and other cyanobacterial groups and **(D)** major eukaryotic phytoplankton lineages illustrate broad spatiotemporal dynamics of phytoplankton diversity and distribution in surface waters. Integrated monthly data from 1991 to 2004 at the BATS site after adjusting to the month of the maximum mixed layer depth (month 0). **(E)** Hierarchical clustering analysis with Bray-Curtis dissimilarity based on the relative abundance of plastid amplicons after assignment to broad taxonomic groups alongside cyanobacterial amplicons in surface waters. Approximately unbiased (AU) probability values based on multiscale bootstrap resampling (10,000 replicates) were calculated and expressed as *p*-values (%). The annual transition from the deep mixing (DM) period to early (Early Stratification) and late stratified (Late Stratification) periods is indicated. Error bars represent the standard deviation. The number of samples used (with each having all data types) for developing unweighted means and pooled standard deviation is indicated by *n*.

A spring phytoplankton bloom and subsequent thermal stratification during summer were evident in chl *a* data (Figures 1A,B; Lomas et al., 2013). At BATS, the highest nutrient concentrations in surface waters occur during the period of Deep Mixing (DM), which helps initiate the bloom, while nutrient concentrations are low during the summer months (Steinberg et al., 2001). During summer, nitrate and phosphate concentrations in most samples were below detection limits, which are 30 and 10 nM, respectively. During month 0, defined as the month when deepest mixing occurs, eukaryotic phytoplankton contributed 56 ± 17% (Figure 1C) and 59 ± 24% (Supplementary Figure S1A) of total phytoplankton amplicons at the surface and at depth, respectively. More generally, in winter/spring (−1 to +5), eukaryotes contributed a higher proportion (44 ± 19%) of photosynthetic amplicons relative to months +6 to +10 (12 ± 8%, *p* < 0.0001, two-tailed Mann-Whitney *U*-test). During the latter strongly stratified summer months, *Prochlorococcus* was abundant (Supplementary Table S1), as observed in previous studies based on cell counts (Durand et al., 2001). Thus, eukaryotic phytoplankton amplicon contributions rivaled those of cyanobacteria (*Synechococcus* and *Prochlorococcus* together; Figure 1C) during the winter/spring months, but were much lower during the period of most intense stratification, as reported previously (Treusch et al., 2012).

Average contributions of broad eukaryotic phytoplankton groups, i.e., stramenopiles, haptophytes, prasinophytes (Viridiplantae) and cryptophytes, to total plastid amplicons varied over the year, especially at the surface (Figure 1D and Supplementary Figure S1B). To identify patterns related to seasonal changes, we performed hierarchical clustering on the relative abundances of groups in surface amplicon data. This resulted in three statistically supported groupings, ‘Deep Mixing (DM)’, ‘Early Stratification’ and ‘Late Stratification’, comprised of month −1 and 0, month +1 to +5 and month +6 to +10, respectively (Figure 1E). During DM, contributions were relatively even throughout the water column. Viridiplantae algae (largely prasinophytes) formed 55 ± 19% (surface) and 53 ± 25% (at depth) of the plastid amplicons and stramenopiles formed 23 ± 15% (surface) and 25 ± 17% (at depth). Haptophytes showed lower but more consistent relative contributions throughout the year, forming 23 ± 8% (23 ± 8%, surface; 22 ± 9%, depth) of plastid amplicons. During Early Stratification (+1 to +5) the community shifted such that stramenopiles comprised about half of plastid amplicons at the surface (57 ± 12%) and developing DCM (53 ± 7%), and had similar contributions during Late Stratification (58 ± 9%, surface; 50 ± 10%, DCM; Figure 1D, Supplementary Figure S1B, Supplementary Table S2). These variations raised questions about whether the stramenopile contributions came from a few taxa, with changes in relative amplicon abundances being a byproduct of dynamics of other phytoplankton groups, or potentially shifts within stramenopile community structure. Therefore, we developed reference phylogenetic trees based on near full-length 16S rRNA gene sequences to further resolve stramenopile taxa.

### Stramenopiles at BATS

Eleven photosynthetic classes were delineated in the near-full length 16S stramenopile plastid reference tree developed herein (Supplementary Figure S2). All 10 known photosynthetic stramenopile classes (Andersen, 2004) were recovered with statistical support alongside a new clade, containing only environmental sequences in a supported position adjacent to bolidophytes, that likely represents an unrecognized class.

**FIGURE 2:**
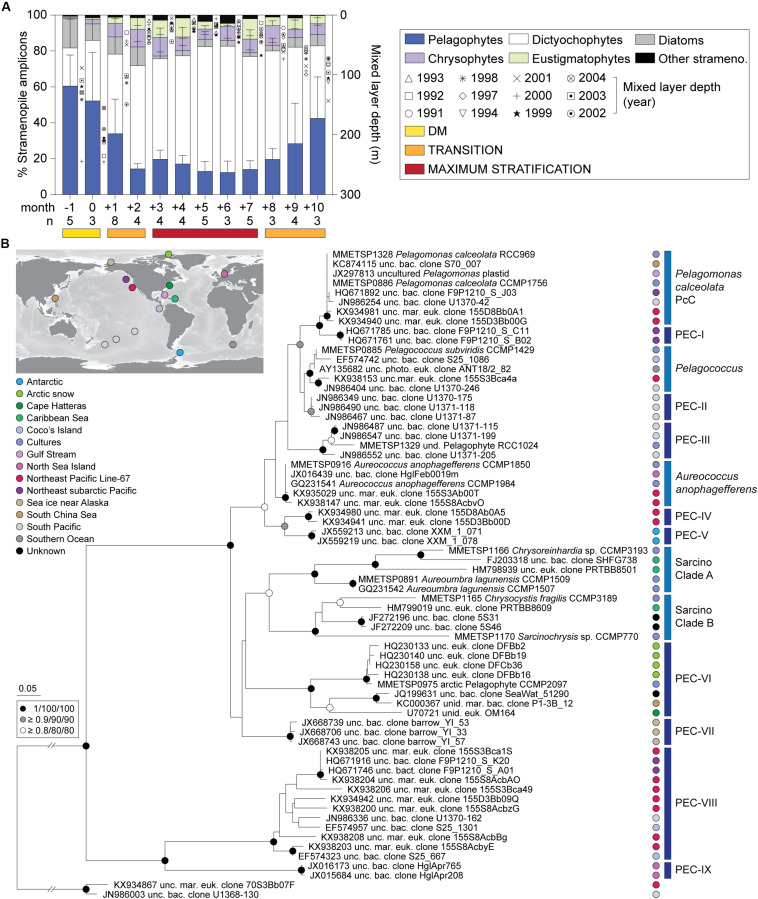
Photosynthetic stramenopile class distributions in the northwestern Sargasso Sea and reference trees for finer-scale analyses. **(A)** Photosynthetic stramenopile distributions expressed as a percentage of amplicons phylogenetically assigned to stramenopile classes at BATS surface (0.3–7.6 m). Error bars represent the standard deviation and ‘n’ indicates the number of samples used for the analysis. Phylogenetic reference trees were developed to then resolve clades within the **(B)** pelagophytes and dictyochophytes (Supplementary Figure S5) using near full-length 16S rRNA gene sequences. For pelagophytes, sequences came from nine cultured species, 56 environmental clones and two dictyochophyte sequences as an outgroup. The tree was constructed with Maximum Likelihood inference (RAxML) under the gamma corrected GTR model of evolution with 1,000 bootstrap replicates. Additional phylogenetic reconstructions were performed with PhyML and MrBayes and the node statistical supports are indicated. Five known clades were resolved and previously unrecognized nine additional clades were identified Pelagophyte Environmental Clades I-IX (PEC-I to PEC-IX). These were primarily comprised of environmental sequences. Colors indicate oceanic region of origin.

Dictyochophytes dominated surface stramenopile amplicons during both the Early and Late Stratification periods, but showed lower, more variable percentages during DM (Figure 2A). Pelagophytes were also notable during these periods and other groups formed a smaller, more variable proportion of stramenopile amplicons. Hierarchical clustering using stramenopile classes alone delineated the DM clearly, as well as a transition into and out of the stratified period, bringing together months +1, +2, +8, +9 and +10 (termed Transitional), separate from months +3 to +7 (Maximum Stratified; Supplementary Figure S3). This grouping gained modest statistical support using pvclust, but not by similarity profile analysis (SIMPROF, *p* > 0.05). Thus, a slightly different seasonal organization of the stramenopile community (albeit with weak statistical support) was observed versus that based on all phytoplankton amplicons (Figure 1E). The stramenopile-based sample clustering corresponded to distinct temperature ranges during DM (20 ± 1°C), Transitional (22 ± 2°C) and Maximum Stratified (26 ± 2°C; *p* < 0.0001, one-way ANOVA with Tukey’s post-hoc test; Supplementary Figure S3).

Diatom relative abundances, as a fraction of stramenopile amplicons, were low and highly variable at the surface: 13 ± 13%, DM; 7 ± 10%, Transitional; and 3 ± 3%, Maximum Stratified period (Figure 2A) as well as at depth (Supplementary Figure S1C, Supplementary Table S3, and Supplementary Data S1). Other groups were typically < 5% of stramenopile amplicons, except chrysophytes (surface; 8 ± 5%, Transitional; 8 ± 5%, Maximum Stratified) and eustigmatophytes (surface; 5 ± 6%, Transitional; 7 ± 7%, Maximum Stratified). Overall, among all stramenopiles recovered, dictyochophytes and pelagophytes dominated amplicon contributions over all three seasonally influenced periods.

Dictyochophytes were most important in surface waters during low nutrient periods, specifically Transitional (51 ± 19%) and Maximum Stratified (64 ± 12%) (Figure 2A and Supplementary Table S3). They formed lower and more variable contributions during DM (28 ± 26%) and their contributions varied, similarly, at depth (40–80 m) during DM and in the DCM as the year progressed (Supplementary Figure S1C). Pelagophyte surface contributions trended opposite to dictyochophytes forming 56 ± 23% (DM) of stramenopile amplicons and dropping to 28 ± 20% and 15 ± 6% in the Transitional and Maximum Stratified periods, respectively (*p* < 0.0001, two-tailed Mann-Whitney *U*-test). Pelagophyte contributions at depth were more consistent year round (54 ± 23%; Supplementary Figure S1C).

These overall trends were upheld at multiple depths in the subset of samples having sufficient sequencing depth for plastid analysis (Supplementary Figure S4A). During DM, pelagophytes dominated stramenopile amplicons throughout the water column (63 ± 16%). The exception was from single samples (160 m, −1; 120 m, 0) when diatoms (e.g., *Chaetoceros*, *Thalassiosira* and unidentified species) were nearly as high, although low overall plastid amplicon numbers suggest few eukaryotic phytoplankton cells were present. Dictyochophytes were the dominant stramenopiles throughout the upper 80 m during the Transitional (43 ± 27%) and Maximum Stratified (59 ± 23%) periods, but generally decreased alongside increased pelagophyte relative abundances below 80 m.

### An Expanded Phylogeny of Pelagophytes and Dictyochophytes

There is a growing literature on the ecological importance of pelagophytes in the ocean (Man-Aharonovich et al., 2010; Worden et al., 2012; Dupont et al., 2015), however, less is known about dictyochophyte distributions and diversity. We examined both these groups at higher phylogenetic resolution. Phylogenetic reconstruction of dictyochophyte 16S rRNA gene sequences delineated 20 clades, including 11 not previously reported and termed here Dictyochophyte Environmental Clades, DEC-I to DEC-VIII (Supplementary Figure S5) and *Florenciella* Clades FC-I to FC-III. All of these clades lack cultured representatives, but FC have high nucleotide identity to *Florenciella parvula*, which forms a separate clade (termed here FpC, *Florenciella parvula* Clade). DEC and FC contained sequences from surface ocean samples collected in the eastern North Pacific, South Pacific, South China Sea, and other habitats, such as the microbial mats from the hypersaline region in Guerrero Negro, Mexico (DEC-V) and the Puerto Rico Trench at 6000 m (DEC-VIII), indicating they are widely distributed and potentially are exported to deep waters.

The reconstruction of pelagophyte 16S rRNA gene sequences resolved the five known pelagophyte clades (Wetherbee et al., 2015), as well as nine undescribed pelagophyte clades, termed here Pelagophyte Environmental Clades, PEC-I to PEC-IX (Figure 2B). Some were solely comprised of sequences from a specific region/study, such as PEC-I (northeast subarctic Pacific Ocean), PEC-II (South Pacific), PEC-IV (ENP), PEC-V (Antarctic), PEC-VII (sea ice near Alaska) and PEC-IX (North Sea near Helgoland).

### Diversity and Seasonality of Pelagophytes at BATS

**FIGURE 3:**
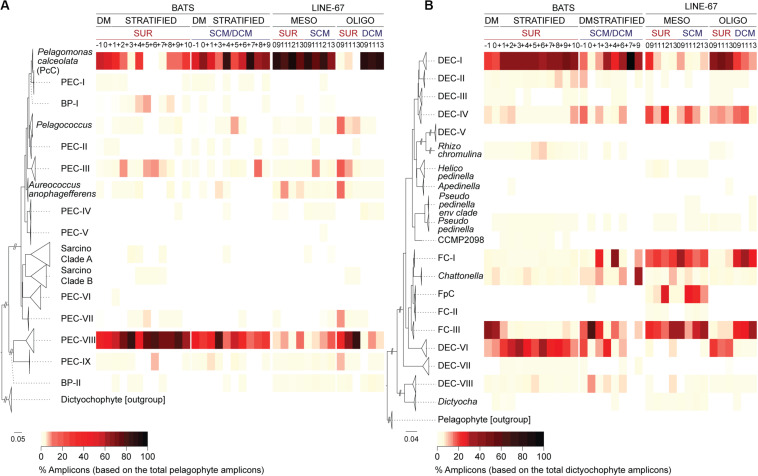
Diversity and distributions of newly resolved dictyochophyte and pelagophyte clades at BATS and in the ENP. High-taxonomic resolution of **(A)** pelagophyte and **(B)** dictyochophyte distributions at the surface (SUR) and depth (SCM/DCM) at BATS and ENP. Reference trees to the left of each panel indicate clades. Heatmaps show the percentage of amplicons relative to those of pelagophytes **(A)** and dictyochophytes **(B)** during each BATS month or ENP samples (vertical columns; see Figure 4 for additional details on ENP samples). Bootstrap support and phylogenetic inferences are as detailed in Figure 2B and Supplementary Figure S5. ENP samples are partitioned based on standing stock nutrient concentrations which were considered mesotrophic (MESO) or oligotrophic (OLIGO) (Supplementary Table S5).

We detected pelagophyte amplicons at BATS belonging to 13 of the 14 clades, including all the uncultured clades formed of full-length sequences from a single region (Figures 2B, 3A). Most clades contributed < 1% of the total pelagophyte amplicons, apart from PEC-V (the one clade not detected in BATS data). By contrast, *P. calceolata* Clade (hereafter PcC) formed 56 ± 23% (surface) and 62 ± 14% (at depth) of pelagophyte amplicons during DM and at the DCM later in the year (59 ± 38%). PcC were low and variable at the surface during the Transitional period (22 ± 21%) and rare during the Maximum Stratified period (1 ± 5%).

Pelagophyte Environmental Clades-VIII contributed relatively high percentages of pelagophyte amplicons during DM at the surface (41 ± 23%) and more so during stratified periods (70 ± 19%, Transitional; 80 ± 16%, Maximum Stratified at the surface; Figure 3A). These trends were upheld when multiple depths were examined for months with deeper amplicon data (−1, 0, +4 and +6; Supplementary Figure S4B and Supplementary Table S4). Additionally, PEC-III and a group of basal sequences (BP-I) were also relatively abundant in Maximum Stratified surface waters (Figure 3A). Overall, PcC and uncultured PEC-VIII had the highest relative abundances among pelagophytes at BATS, but BP-I and PEC-III, also rose in prominence during intense stratification (Figure 3A).

### Uncultured Dictyochophytes in the Sargasso Sea

A diverse set of uncultured dictyochophyte clades exhibited seasonal variations. Dictyochophytes formed 35 ± 10% of all plastid amplicons in BATS Maximum Stratified surface waters (Figure 2A). Assignment of amplicons to distinct clades showed that uncultured groups DEC-I and DEC-VI were prevalent during this period while FC-III appeared more important during DM throughout the water column (Figure 3B). DEC-I contributed the most dictyochophyte amplicons at the surface during Transitional (43 ± 11%) and Maximum Stratified periods (39 ± 11%), and at the DCM during the latter (49 ± 25%). DEC-VI was also notable at the surface during Transitional (23 ± 14%) and Maximum Stratified (26 ± 15%), but less important at depth. Corresponding FC-III contributions were minor except during DM, when they formed 38 ± 27% (surface) and 39 ± 16% (at depth) of total dictyochophyte amplicons.

These patterns were generally maintained in 19 vertical profiles examined. During the DM period FC-III formed the greatest percentage of dictyochophyte amplicons throughout the photic zone (∼140 m; Supplementary Figure S4C). In contrast, during the Transitional and Maximum Stratified periods, DEC-I had the highest relative contributions at the surface, but at the DCM (ranging from 40–120 m) it was rivaled by other clades. Thus, uncultured environmental groups comprised most of the dictyochophyte amplicons at BATS (e.g., in surface waters 92 ± 4%, DM; 92 ± 5%, Transitional; 85 ± 4%, Maximum Stratified). Further, they contributed a large proportion of the total eukaryotic phytoplankton amplicons in surface waters, especially during Transitional (30 ± 14%) and Maximum Stratified (30 ± 10%) periods. This indicates that uncultured dictyochophytes form a large portion of the eukaryotic phytoplankton that persist at BATS during oligotrophic periods, when *Prochlorococcus* typically dominates cell counts.

### Comparisons With ENP Communities

**FIGURE 4:**
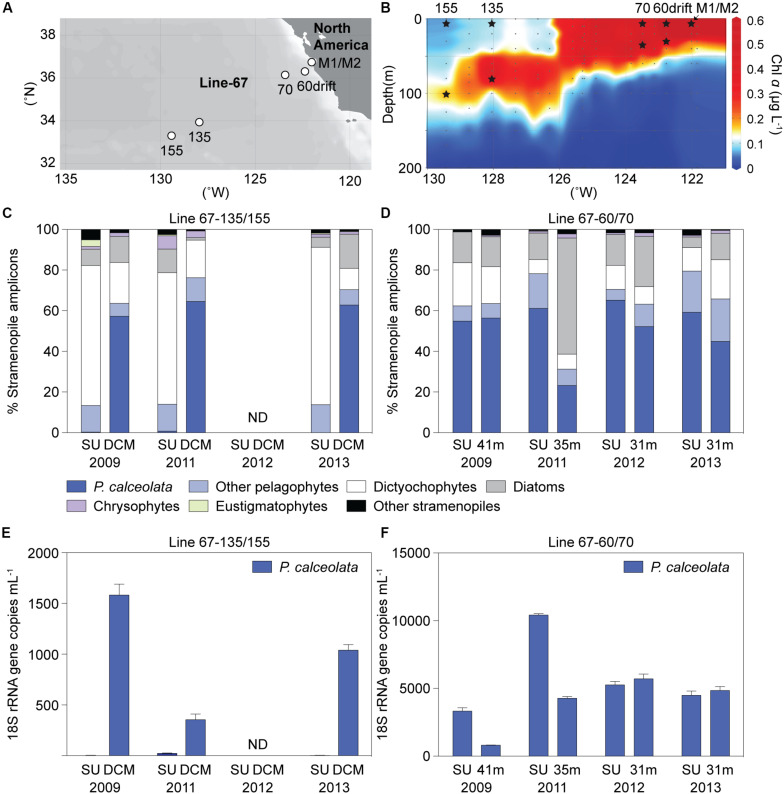
ENP **(A)** stations and **(B)** chlorophyll *a* depth profile in 2009 with sampling for DNA indicated by stars (Stations 67-60drift and 67-70, mesotrophic sites; Stations 67-135 and 67-155, oligotrophic sites). Relative abundance of stramenopile V1-V2 16S rRNA gene amplicons assigned to the *Pelagomonas calceolata* Clade or other groups in Line-67 samples. **(C)** Taxon distributions at sites with well stratified water columns where nitrate was 0.02 and 0.08 μM (plus 1 sample < 30 nM detection limit) at the surface and 0.05 to 0.3 μM (0.17 ± 0.13 μM, *n* = 3) in the deep chlorophyll maximum (DCM). **(D)** Distributions in surface (SU, 2–14 m) or near the base of the photic zone (31-41 m) at mesotrophic sites. **(E,F)** *P. calceolata* 18S rRNA gene copies ml^–1^ enumerated by qPCR in the same samples as analyzed for panels C, D. Note the different scale on the *y*-axis in **(E)** and **(F)**. ND: no data. Error bars represent the standard deviation of three technical replicates.

The Sargasso Sea results indicated the potential importance of diverse uncultured dictyochophyte clades under oligotrophic conditions and a phylogenetically narrower range of pelagophyte clades at the nutricline of stratified water columns. We therefore next examined ENP communities at two well characterized (Monier et al., 2012; Sudek et al., 2015; Simmons et al., 2016; Limardo et al., 2017) stratified oligotrophic sites (termed “OLIGO”), specifically Station 67-135 and 67-155, based on 3 surface and 3 DCM samples (Figures 4A,B and Supplementary Table S5). We also characterized samples from a more mesotrophic region (termed MESO), based on four surface and four SCM samples collected at Stations 67-60drift and 67-70. Here, we distinguish between periods or sites exhibiting a deep chlorophyll maximum (DCM), as seen in oligotrophic gyres, typically located between 80 to 130 m, depending on the season and system (Karl and Church, 2014), and subsurface chlorophyll maxima (SCM), that often occur in the upper 40 m of more nutrient-rich sites.

Oligotrophic nitrate concentrations at the surface ranged from < 30 nM (detection limit) to 80 nM, and from 50 to 300 nM (*n* = 3) at the DCM, while phosphate was much higher (e.g., > 340 nM, Supplementary Table S5). Similar to BATS, *Prochlorococcus* dominated photosynthetic amplicons at ENP OLIGO sites, forming 92 ± 2% and 84 ± 6% at the surface and DCM phytoplankton amplicons, respectively (Supplementary Figure S6A). Eukaryotic phytoplankton amplicon contributions (7 ± 2%, surface; 15 ± 6%, DCM) followed and were higher than *Synechococcus* (0.9 ± 0.0%, surface; 0.2 ± 0.1%, DCM). Stramenopiles comprised 69 ± 5% and 57 ± 7% of plastid amplicons, at the surface and DCM, respectively, appearing to dominate eukaryotic phytoplankton amplicons in the oligotrophic stations (Supplementary Figure S6C).

At ENP MESO stations, eukaryotic phytoplankton amplicons were more abundant at the surface (59 ± 18%) and SCM (65 ± 21%) than cyanobacteria (Supplementary Figure S6B). In the four MESO SCM samples stramenopiles averaged 45 ± 8% of total plastid amplicons. During the more nutrient rich ENP MESO sample periods (2011 and 2012, 2.05 ± 0.66 μM; surface NO_3_^–^) stramenopile contributions were 22 ± 9% in surface waters, similar to BATS during the DM period (23 ± 15%). In 2009 and 2013, when ENP MESO surface nitrate was lower (0.33 ± 0.10 μM), stramenopile contributions were stronger, more akin to oligotrophic waters (Supplementary Figure S6D).

Among stramenopiles, dictyochophyte amplicons were the most highly represented in OLIGO surface samples (70 ± 6%). They formed 13 ± 6% of stramenopile amplicons at the MESO surface and SCM, similar to the OLIGO DCM (16 ± 5%) (Figures 4C,D). Pelagophytes had the highest relative abundances within stramenopile amplicons in OLIGO DCM samples (70 ± 6%). They formed 73 ± 8% and 56 ± 17% of stramenopile amplicons at the MESO surface and SCM, respectively, while much lower in OLIGO surface samples (14 ± 0%).

### ENP Dictyochophyte and Pelagophyte Diversity Patterns

We next evaluated the community structure of dictyochophytes and pelagophytes in the ENP. Among dictyochophytes, DEC-I (43 ± 3%) and DEC-VI (19 ± 3%) were notable in OLIGO surface samples (Figure 3B) as observed in BATS surface samples during the Transitional and Maximum Stratified periods. At the ENP SCM and DCM, FC-III and FC-I contributions were higher than either DEC-I and DEC-VI (Figure 3B; 29 ± 6%, FC-III; 28 ± 3%, FC-I). FC-III was also prominent in ENP mesotrophic waters, representing 24 ± 8% (surface) and 27 ± 10% (SCM) of total dictyochophyte amplicons (Figure 3B). Contributions from other *Florenciella* clades to dictyochophyte amplicons were also notable in ENP MESO samples, such as FC-I (19 ± 2%, surface; 22 ± 6%, SCM) as well as FpC (12 ± 11%, surface; 17 ± 9% SCM) which was not observed in ENP OLIGO samples. The latter two *Florenciella* clades were rare in BATS amplicon data. Another clade that appeared to have greater importance in the ENP relative to BATS was DEC-IV, which formed 15 ± 9% (surface) and 12 ± 6% (SCM) of MESO dictyochophyte amplicons.

With respect to pelagophytes, PcC was the most prominent clade in ENP mesotrophic waters, forming 83 ± 8% (surface) and 78 ± 9% (SCM) of the total pelagophyte amplicons (Figure 3A). It represented 24 ± 13% (surface) and 20 ± 8% (SCM) of total plastid amplicons. PEC-VIII formed only 8 ± 6% (surface) and 8 ± 7% (SCM) MESO pelagophyte amplicons, similar to OLIGO DCM (6 ± 3%) levels. PcC contributions to total pelagophyte amplicons were high (88 ± 3%) in OLIGO DCM samples but dropped to 2 ± 3% in surface waters, concurrent with an increase in PEC-VIII (63 ± 26%). Amplicons assigned to the clade containing *A. anophagefferens* and PEC-III were ≤ 10% on average in all ENP OLIGO and MESO samples. Other environmental pelagophyte clades were detected and contributed < 1% or, for *Pelagococcus*, PEC-VII and PEC-IX, averaged < 10% of total pelagophyte amplicons in oligotrophic surface waters.

To further validate results derived from amplicon relative abundance patterns we quantified abundance using qPCR (Figures 4E,F). Secondly, having noticed that dictyochophytes reach their highest relative abundances during the stratified conditions, when the oligotroph *Prochlorococcus* exhibits its greatest numerical abundances, we examined the potential trophic modes and evolution of uncultured dictyochophytes for clues to their success in nutrient-deplete systems (Durand et al., 2001; Malmstrom et al., 2010).

### Relationship Between Relative Amplicon Abundance and Quantitative Analysis

Our analyses identified previously unrecognized environmental clades and provided insights into dynamics of phytoplankton community structure. However, patterns in relative amplicon abundance are strongly influenced by the interplay between a taxon’s own abundance and changing abundances of other taxa. Moreover, differences in gene copy number between different phytoplankton groups remain unconstrained (Demir-Hilton et al., 2011; Limardo et al., 2017). Therefore, using PcC as a case study, we quantified *P. calceolata* abundance in Line-67 samples using an 18S rRNA qPCR primer-probe set developed herein (Supplementary Tables S6, S7). *P. calceolata* was selected because of its importance in samples with higher nutrient concentrations at both BATS and the ENP, and in other semi-quantitative studies (Worden et al., 2012; Dupont et al., 2015). The abundance of *P. calceolata* varied considerably in mesotrophic samples over the four years: 5,882 ± 2,855 (surface) and 3,917 ± 1,976 (SCM) 18S rRNA gene copies ml^–1^ (range from 792 to 10,596 18S rRNA gene copies ml^–1^). It had low abundance in OLIGO surface samples (9 ± 11 copies ml^–1^; range from below detection to 26 copies ml^–1^; Figures 4E,F), in agreement with amplicon analyses. Its abundance was higher at the OLIGO DCM (992 ± 538 copies ml^–1^; range 289 to 1,665 copies ml^–1^), supporting its prominence in DCM amplicon data (Figures 4C,D).

Amplicon relative abundance data did sometimes mask differences observed in quantitative abundance data. For example in 2009, surface and SCM mesotrophic samples showed abundances of 3,334 ± 425 and 819 ± 24 18S rRNA gene copies ml^–1^, respectively (Figure 4F; SU vs. 41 m), whereas relative abundance of *P. calceolata* in stramenopile amplicons was almost equal (55%, surface; 56%, SCM) between these samples (Figure 4D), and only moderately different relative to all plastid amplicons (40%, surface; 30% SCM). Additionally, while there was little difference in absolute abundance between 2011 and 2012 mesotrophic SCM samples (Figure 4F; 35 m in 2011 vs. 31 m in 2012), amplicon data suggested there were large changes (Figure 4D), which, in light of the qPCR data, now appear to have been driven by increased abundance of other phytoplankton taxa in the 2011 sample, reducing relative pelagophyte contributions.

### Dictyochophyte Dynamics and Activity Expose a Predatory Mixotroph Signature

The distribution patterns of environmental dictyochophyte clades showed greater prominence during conditions of low nutrient availability where predatory mixotrophic strategy might be advantageous (Edwards, 2019; Wilken et al., 2019). We therefore used single-cell sorting that targeted eukaryotic cells (Cuvelier et al., 2010) with stained food vacuoles to try to recover mixotrophic dictyochophytes in ENP waters (Figures 4A,B; Stations M1 and M2; Supplementary Figure S7A). Several uncultured dictyochophytes were recovered based on 16S V4 amplicon sequencing (Supplementary Figure S7B). Sequencing of single-cell sorts revealed a dictyochophyte representing DEC-IV (DEC-IV sort, 100% nt identity), a *Pseudopedinella*-like dictyochophyte (*Pseudopedinella*-like sort, 97% nt identity), two Pedinellales-like sorts and seven other more divergent dictyochophytes that had < 95% nucleotide identity to reference sequences, based on analysis of the V4 region of the 16S rRNA gene.

The seven more divergent sorted dictyochophytes formed two distinct environmental clades. One, DEC-IX comprised four sorted cells (Supplementary Figure S7B) for which we then sequenced the complete 16S rRNA gene in order to better resolve their phylogeny relative to other dictyochophytes. The reconstruction based on near-full length 16S sequences indicates that DEC-IX is basal to multiple other environmental sequences and clades (Supplementary Figure S8). The other three more divergent sorted cells grouped together, forming the distinct clade DEC-X (Supplementary Figure S7B). DEC-X cells were unlike other sorted cells in having minimal chl *a* fluorescence (i.e., near the baseline in the chl *a* channel), indicating they potentially had non-functional remnant plastids (achlorophyllous), or very low chlorophyll content, and hence likely require prey to grow (Supplementary Figure S7A).

We next constructed both full-length 18S rRNA gene and 18S V4 amplicon–based reconstructions for the dictyochophyte sorts (Supplementary Figure S9). All but one 18S amplicon were placed in environmental-only clades. The one exception (termed here CCMP2098-like sort 1) had 100% 18S rRNA gene identity to cultured Pedinellales isolates from the Arctic Ocean, i.e., CCMP2098, RCC2301 and RCC2286. The branching position of the CCMP2098 clade differed between the 18S and 16S rRNA gene reconstructions, but it was consistently separate from *Florenciella*. Unfortunately, incomplete taxon sampling of dictyochophyte (especially the lack of 16S and 18S rRNA genes from the same reference organisms) confounds comparisons of the tree topologies at finer scales.

**FIGURE 5:**
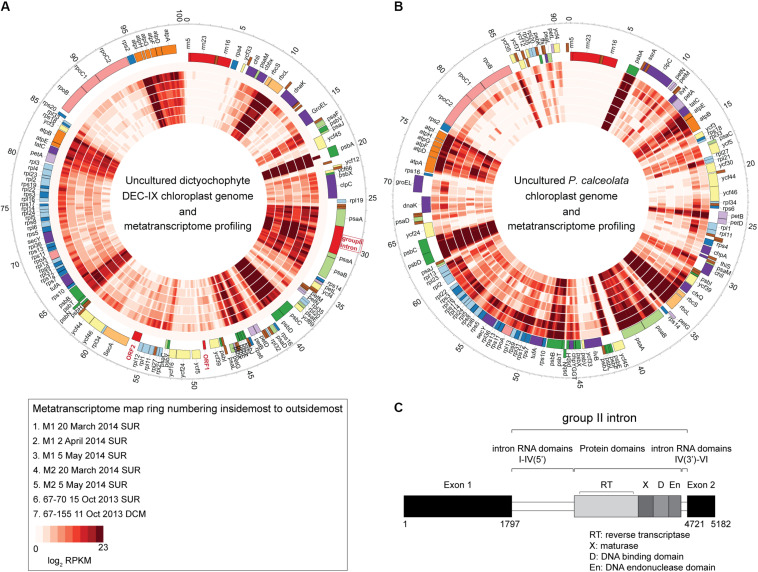
The complete chloroplast genome of **(A)** uncultured dictyochophyte DEC-IX **(B)** and wild *P. calceolata* (Worden et al., 2012), and transcriptional activity in the ENP. Recruitment of metatranscriptomic reads (inner layers) at ≥ 99% nucleotide identity shown as mapped reads binned at 100 bp increments and log2 transformed for the heat plots which reflect the normalized transcripts. While the metatranscriptomes were unreplicated and therefore unsuitable for quantifying expression, qualitatively, similar patterns of expression were observed for genes present in both the DEC-IX and *P. calceolata* chloroplast genomes. All come from surface samples except the 67-155 metatranscriptome (100 m, DCM). Outermost ticks represent kilobase pairs (chloroplast genome size). **(C)** Structure of the *psaA* gene group II intron, showing upstream and downstream exons, intron domains and conserved IEP consisting of a reverse transcriptase (RT), a maturase domain (X), a DNA-binding domain (D) and an endonuclease domain (En).

Given the interesting phylogenetic position of the new DEC-IX clade, as well as the paucity of genomic data from dictyochophytes, we next performed metagenomic sequencing on cells that had both chlorophyll fluorescence and food vacuoles (based on LysoTracker and LysoSensor signals; Supplementary Figure S7A). We assembled DEC-IX’s complete chloroplast genome, which has 100,783 bp (36.9% G+C) and encodes 113 proteins, 28 tRNAs, and an rRNA operon as well as other features (Figure 5A). Compared to *P. calceolata* (Worden et al., 2012), which was also abundant in our amplicon data, the DEC-IX chloroplast genome has two fewer protein encoding genes, 6 additional characterized genes (*psaE*, *psbY*, *ycf66*, *rpl12*, *rpl32* and *rps19*), and additional ORFs, ORF1 and ORF2, of unknown function (Supplementary Data S2).

The DEC-IX plastid genome also has a group II intron (2923 bp) inserted in the *psaA* gene (Figure 5C). The DEC-IX *psaA* gene appears to be intact with no sign of disruption by this intron invasion and was actively expressed in ENP metatranscriptomes (Figure 5A). Based on predicted secondary structure, it is a type IIB intron with six predicted RNA domains (DI-DVI) common to group II introns and contains the conserved specific junction sequences at the boundaries of the introns (5′-GUGYG…AY-3′) (Lambowitz and Zimmerly, 2011). An IEP (Intron Encoded Protein) of 593 amino acids is encoded within the loop of DIV, containing the characteristic domains for reverse transcriptase (RT), maturase (X), DNA binding (D) domain and the H-N-H endonuclease domain (En). Transcripts from *psaA* and the group II intron were identified in the seven ENP metatranscriptomes analyzed by mapping to the chloroplast genome generated herein (Figure 5). Otherwise, major components of PSII from both DEC-IX and *P. calceolata*, encoded by *psbA*, *psbB*, *psbC* and *psbD*, were most highly expressed along the transect, relative to the other chloroplast-encoded genes they contain, as were the RUBISCO small and large subunits *rbcS* and *rbcL* (Figures 5A,B and Supplementary Data S3). Furthermore, the two novel DEC-IX ORFs (ORF1, 276 nt and ORF2, 915 nt) were transcribed. Akin to amplicon relative abundance patterns, higher read numbers mapped to DEC-IX in surface samples, than in the single DCM metatranscriptome sequenced, in which *P. calceolata* recruited many transcripts (Supplementary Data S3).

## Discussion

Here, 16S rDNA-based metabarcoding methods were used to examine phytoplankton diversity in time-series (BATS) and transect (ENP) samples. We captured marked seasonal trend and contributions from photosynthetic stramenopiles, which accounted for up to 73% of the total eukaryotic phytoplankton amplicons in the samples. The high relative abundances of dictyochophytes in oligotrophic waters and pelagophytes in mesotrophic or seasonally nutrient rich waters that we report are a previously unrecognized feature of stramenopile ecology. We focused on the unexpected observation that dictyochophytes, a stramenopile class largely represented by silicoflagellates in culture (Eikrem et al., 2004), reached their highest relative abundances in the most oligotrophic samples. High precision in phylogenetic identification was achieved by building a well curated backbone tree from plastid sequences, enabling us to identify transitions among numerous uncultured clades that had not clearly emerged from previous studies.

### Phytoplankton Successional Patterns

Our amplicon analyses demonstrated that prasinophytes bloom during the BATS DM period, consistent with results from an earlier T-RFLP study (Treusch et al., 2012). These blooms were followed by a shift to eukaryotic communities dominated by stramenopiles as the water column became stratified and nutrients decreased (Figure 1D). At the coarse level of phylogenetic supergroups, eukaryotic phytoplankton did not shift dramatically after DM, unlike cyanobacteria, where *Prochlorococcus* dominance was most pronounced late in the stratified period (Figure 1C). However, the high-resolution analysis of eukaryotic plastids captured previously unobserved seasonal shifts in the stramenopile community at BATS. During the DM period, pelagophytes, specifically PcC, were the dominant stramenopiles. In the transition to stratification, we observed reduced percentages of pelagophyte amplicons and increased contributions from dictyochophytes. During the stratified period, when stramenopiles were the most prominent eukaryotic phytoplankton, surface waters were dominated by dictyochophytes, while pelagophytes exhibited higher relative contributions in DCM communities (Figure 2A and Supplementary Figure S1). Diatoms, the most well-studied photosynthetic taxa among stramenopiles, rarely reached high relative abundances in our 10 year ∼monthly sample set and their variability (0 to 37% of stramenopile amplicons) appeared to be driven by punctuated, ephemeral blooms occurring or captured in some years and not others.

When the highly resolved genetic data was synopsized to provide a qualitative overview of the biogeography of the major phyla in the ENP, distinct spatial distributions were identified across environmental gradients, from oligotrophic ocean dominated by *Prochlorococcus*, to a mesotrophic region with increased signals from *Synechococcus* and eukaryotic phytoplankton. While not a focus in this paper, our primers clearly recovered diatoms as these phytoplankton were abundant in an ENP coastal sample (Supplementary Figure S10). Among eukaryotic phytoplankton at the mesotrophic and oligotrophic sites, stramenopiles and prasinophytes were the two most abundant phyla (Supplementary Figure S6). The seasonal analysis of BATS revealed that large overall contributions of stramenopiles at the surface and DCM during stratified periods were driven by pelagophytes and dictyochophytes, paralleling features of community structure observed in OLIGO ENP samples. In contrast, the ENP mesotrophic region showed interannual variability at the surface, with stramenopile dominance during 2009 and 2013, versus prasinophyte dominance during 2011 and 2012. Mesotrophic ENP stations were dominated by pelagophyte amplicons throughout the photic zone (64 ± 15%), similar to what was observed during the DM period at BATS (60 ± 21%). Indeed, hierarchical clustering based on stramenopile classes using BATS and ENP surface amplicon data, for which we had the greatest statistical power, showed the BATS DM period clustering with mesotrophic ENP sites and the stratified periods with oligotrophic ENP sites (Supplementary Figure S11). Additionally, contributions at depth were remarkably stable over the regimes and sites studied, although stramenopiles in the ENP MESO SCM were notably higher than at BATS during DM period (Supplementary Figure S6D). We note that the ENP MESO SCM has measurable nutrients while at BATS during the DM period nutrients are frequently below detection. As a whole, stramenopiles contributed less to the overall community relative to other phytoplankton groups, particularly prasinophytes and cyanobacteria, in BATS DM and ENP MESO samples. However, collectively, the dominant stramenopile lineages across seasonal and combined regime averages for BATS, and the ENP OLIGO and MESO sites, were dictyochophytes and pelagophytes.

High-performance liquid chromatography-based studies have reported that pelagophytes and haptophytes are important at BATS, with abundance peaks occurring during DM and at the DCM (Andersen et al., 1996; Goericke, 1998; Haidar and Thierstein, 2001; Steinberg et al., 2001). Additionally, prasinophytes were shown by qPCR to have high numerical abundances during spring and even to bloom relative to other taxa during DM based on T-RFLP analyses (Treusch et al., 2012), similar to our observations. Silicoflagellates have also been reported at BATS, in a microscopy-based study on haptophytes (Haidar and Thierstein, 2001). Our studies resolve phytoplankton molecular diversity and seasonal patterns, and demonstrate a previously unrecognized nutrient-linked seasonal switch between pelagophytes and dictyochophytes.

Our findings demonstrate that dictyochophyte amplicons form the greatest percentage of those from eukaryotic phytoplankton, and dominate stramenopile amplicons in surface waters throughout the stratified period at BATS (Figure 2A) and in oligotrophic surface waters of the North Pacific Gyre (Figure 4C). These results are different from conclusions on important taxa in prior studies and it is possible that the seasonal importance of dictyochophytes observed herein has gone undetected in some studies, especially HPLC-based studies, because their pigments overlap with those of better-studied taxa, such as diatoms. Cultured dictyochophytes contain Chl *a* and *c*, and fucoxanthin as the main carotenoid, a carotenoid also found in diatoms, haptophytes and chrysophytes, alongside one acylfucoxanthin derivative, 19′-butanoyloxyfucoxanthin [19′-but], which is generally considered a specific marker for pelagophytes (Daugbjerg and Henriksen, 2001; Eikrem et al., 2004; Chang et al., 2012). Thus, cultured dictyochophytes exhibit pigments typically considered as markers for other, better-studied phytoplankton classes (Daugbjerg and Henriksen, 2001). Variations in pigment composition among the few cultured dictyochophyte groups further confound field sample interpretation. For example, *Dictyocha speculum* and *Vicicitus globosus* possess 19′-hexanoyloxyfucoxanthin [19′-hex], a major pigment in haptophytes (Daugbjerg and Henriksen, 2001; Chang et al., 2012), whereas pedinellids and *Rhizochromulina* have 19′-but and lack 19′-hex (Daugbjerg and Henriksen, 2001). Our analyses indicate that dictyochophytes likely contribute significantly to the oceanic 19′-but and 19′-hex pigment pool, and that their contributions were previously miss-assigned to pelagophytes and haptophytes. Indeed, as outlined above, prior HPLC studies at BATS indicated haptophytes and pelagophytes were the most abundant eukaryotic phytoplankton groups (Andersen et al., 1996; Goericke, 1998; Steinberg et al., 2001). Given that our primers clearly recovered pelagophytes and haptophytes, it seems likely that the importance of dictyochophytes in eukaryotic phytoplankton communities has been significantly underestimated. Collectively, these results shift our view of how different phytoplankton groups contribute at BATS, and in the ENP, and identify potential shifts in trophic modes among eukaryotic phytoplankton (see below).

### Extensive Molecular Diversity of Photosynthetic Stramenopiles

In addition to the broad trends discussed above, phylogenetic reconstructions of near full-length 16S rRNA gene sequences revealed numerous previously unrecognized clades within both the pelagophyte (Figure 2B) and dictyochophyte (Supplementary Figure S5) classes. Moreover, we identified a novel eukaryotic (plastid-based) clade that is a sister of bolidophytes and may represent a new stramenopile class (Supplementary Figure S2).

High resolution phylogenetic comparisons of stramenopiles between BATS and the ENP indicated that several dictyochophytes clades, DEC-I, DEC-VI and FC-III, were prominent at BATS and ENP (Figure 3B). These groups also drove the trends seen for the overall class. Among dictyochophyte amplicons, DEC-I and DEC-VI were prevalent during the Transitional and Maximum Stratified periods in BATS surface waters and oligotrophic ENP surface waters, while FC-III was prominent during DM period and the DCM of the ENP oligotrophic site. Different patterns were observed for other clades, such as FC-I and FpC, which were barely detected at BATS and more abundant along ENP Line-67. Similarly, increased contributions from DEC-IV were found in ENP samples (Figure 3B). Among pelagophytes PcC showed high relative abundances at depth throughout the year at BATS and on Line-67 (mesotrophic and oligotrophc stations), whereas it had much lower relative abundances at the surface during the BATS stratified period and on Line-67 at the most oligotrophic sites (Figure 3A). In contrast PEC-VIII showed high relative abundances among pelagophytes at BATS throughout the year regardless of depth or period, but in the ENP only exhibited high abundance at the surface of the oligotrophic site. Our results also indicate that current phytoplankton isolation enrichment methods are not suited to recovering these important taxa, as frequently discussed for heterotrophic protists (Keeling et al., 2014; Needham et al., 2019), or that insufficient effort has been made to recover cultures from oligotrophic sites. In fact, just one dictyochophyte clade observed in our samples is represented in culture, and it exhibited higher relative abundances in more nutrient-rich waters, whereas the key clades found in oligotrophic samples remain uncultured.

### Distinct Pelagophyte Clades and Contributions During More Nutrient Rich Periods

The first described pelagophyte, *Pelagomonas*, is considered a cosmopolitan genus that is frequently isolated from sea water (Andersen et al., 1993; Andersen et al., 1996). Recently, metagenomic approaches revealed their presence in SCM communities, but not in the surface (Dupont et al., 2015). Our results clearly demonstrated that *P. calceolata* is among the major phytoplankton taxa in SCM/DCM communities, at least based on relative amplicon abundances. This corresponds well with results from a metagenomic mapping study using the *P. calceolata* chloroplast genome, which showed it being broadly distributed (Worden et al., 2012). Additional metatranscriptomic (Dupont et al., 2015; Kolody et al., 2019) and qPCR (herein) studies correspond relatively well with our amplicon-based inferences. Interestingly, a metatranscriptomic study in the oligotrophic North Pacific suggested that *P. calceolata* dominated nitrate assimilation in the SCM, but was experiencing nutrient stress in surface waters (Dupont et al., 2015). Here, during the BATS DM period, when vertical mixing brings nutrients to the surface, *P. calceolata* showed higher relative abundances at the surface than throughout the rest of the year (Figure 3A), supporting the idea that this species relies on nitrate as a primary nitrogen source. In terms of overall abundances, while *P. calceolata* was detected by qPCR and formed a notable percentage of stramenopile amplicons, other eukaryotic phytoplankton comprised the majority of plastid amplicons at BATS during DM and often in the ENP MESO samples (e.g., prasinophytes, up to 79% of plastid amplicons in each setting).

We identified an unknown pelagophyte clade, PEC-VIII, in ENP OLIGO and BATS stratified surface waters where nutrient availability was low relative to other periods and sites (Figure 3A). Reference sequences for PEC-VIII clade come from prior studies in the northeast subarctic Pacific, South Pacific, tropical Pacific near Cocos island, and the ENP (Allers et al., 2013; Yin et al., 2013; Choi et al., 2017), suggesting their broad distribution and potential importance in the phytoplankton communities of oligotrophic surface oceans. At BATS, PEC-VIII was a major contributor at the surface during the stratified period. Phagotrophy has not yet been observed in cultured pelagophytes (Stoecker et al., 2006), however, *Aureococcus* utilizes dissolved organic matter (as likely many phytoplankton do).

### Dictyochophyte Importance in Oligotrophic Surface Oceans

Dictyochophytes have been reported in other molecular surveys of planktonic communities (Shi et al., 2011; de Vargas et al., 2015). A striking finding from our high-resolution phylogenetic analyses beyond the broad molecular diversity of dictyochophytes (Supplementary Figure S5), which has been noted in the Tara Oceans study (de Vargas et al., 2015), is the remarkably strong, reproducible patterns that aligns with low nutrient conditions or potentially co-associated factors (e.g., temperature, Figure 3B). In our samples dictyochophytes formed up to 81% and 77% of stramenopile amplicons in oligotrophic BATS and ENP surface waters, respectively, and, on average, 64 ± 12% during the Maximum Stratified period at BATS, and 70 ± 6% in the ENP OLIGO surface waters (Figures 2A, 4D). Further, the majority of these amplicons came from uncultured dictyochophyte groups that we identify herein.

One factor that could drive plankton composition in oligotrophic waters is vitamin biosynthesis capabilities, since vitamins exhibit low concentrations (Sañudo-Wilhelmy et al., 2012). For example, eukaryotic phytoplankton taxa present at BATS exhibit different configurations of thiamin biosynthetic genes (McRose et al., 2014; Gutowska et al., 2017; Paerl et al., 2017), which led us to investigate available dictyochophyte transcriptome-derived genomic information (Keeling et al., 2014). *Pseudopedinella elastica* CCMP716 and the related undescribed species CCMP2098 have complete thiamin pathways (Supplementary Table S8) and phylogenetically group together with clade DEC-I, the main dictyochophyte clade in stratified waters (Figure 3B). The other dictyochophyte with a complete pathway, *D. speculum* CCMP1381, is never numerically abundant in our samples. The four *Florenciella* transcriptomes in the MMETSP dataset contain only one or no genes from the thiamin pathway (Supplementary Table S8). While absence in transcriptomes is not definitive, it is interesting to note that the environmental *Florenciella*-like clades are most abundant when nutrients are elevated (although vitamin concentrations are not known), such as in BATS surface waters during deep mixing, the DCM, and ENP mesotrophic waters (Figure 3B).

### Biology and Evolution of Dictyochophytes

Most of the clades identified herein lack cultured representatives. Hence, reports of dictyochophyte biology, including reports of predatory, mixotrophic nutritional modes (Sekiguchi et al., 2003), come largely from cultured taxa that belong to phylogenetic clades different from those that dominated at BATS and the ENP (Supplementary Figure S5). Likewise, our knowledge of dictyochophyte pigment composition (Daugbjerg and Henriksen, 2001; Eikrem et al., 2004; Chang et al., 2012), and silicoflagellate cell structure (Henriksen et al., 1993), comes from cultured species. Silicoflagellates have served as paleoecological markers, due to their distinct silicate skeletons (Rigual-Hernandez et al., 2016), and have been shown to be common in nutrient rich, high-latitude environments by HPLC and light microcopy (van de Poll et al., 2018). The Dictyochales order as a whole takes a basal position within the dictyochophyte phylogeny (Supplementary Figure S5). Further, *Florenciella* which holds a less basal position, encodes the well characterized *SIT* gene, a Si transporter, also present in diatoms and several other marine eukaryotic lineages (Marron et al., 2016) and is purportedly a mixotroph based on field SIP data (Frias-Lopez et al., 2009). Because the dominant clades observed at BATS and the ENP lack cultured representatives, it remains unclear whether the capacity to feed via phagocytosis extends across dictyochophyte diversity. However, as with diatoms (Durkin et al., 2016), which were low in relative abundance at our study sites, dictyochophyte silicification has been associated with efficient carbon export (Marron et al., 2016; Rigual-Hernandez et al., 2016).

Just one known predatory mixotrophic stramenopile had a sequenced chloroplast genome, the chrysophyte alga *Ochromonas* CCMP1393 (Supplementary Figure S2) (Sevcikova et al., 2015), until the recent addition of plastid genomes from four cultured dictyochophytes species *D. speculum*, *Rhizochromulina marina*, *F. parvula* and *P. elastica* (Han et al., 2019). We sequenced the complete chloroplast genome of newly identified uncultivated clade DEC-IX from a wild cell. The phylogenetic position of DEC-IX requires further evaluation using phylogenomic approaches, as the tree topology using near full length 16S rRNA gene sequences was supported primarily at terminal nodes (Supplementary Figure S8), and the use of missing positions to place 16S and 18S amplicons (Supplementary Figures S7, S9) also rendered largely unsupported nodes, apart from terminal nodes. The DEC-IX chloroplast genome (100,783 bp) is smaller than that of *Ochromonas* and several other stramenopiles, e.g., diatoms and eustigmatophytes (Sevcikova et al., 2015), and the four dictyochophytes, which range from 108,152 to 140,025 bp. However, it is larger than that of *P. calceolata* (91,306 bp), one of the major players in our study, and other pelagophytes (*A. anophagefferens*, 89,599 bp; *A. lagunensis*, 94,346 bp) (Ong et al., 2010). The numbers of protein encoding genes in the chloroplast genomes of DEC-IX and the four cultured dictyochophytes (Han et al., 2019) are similar. Curiously, both the *D. speculum* and DEC-IX plastid genomes lack the inverted repeats (IRs) present in other dictyochophyte plastid genomes (Han et al., 2019) and many other plastid-bearing taxa (Sabir et al., 2014). *D. speculum* however, has a distinct phylogenetic position from DEC-IX, based on 16S and 18S phylogenies (Supplementary Figures S7–S9).

The group II intron in the *psaA* gene of DEC-IX is relatively rare. *PsaA* encodes a protein critical for binding P700, the primary electron donor of photosystem I. The presence of a mobile genetic element, specifically a group II intron in a *psaA* gene, has previously been reported in the green alga *Chlamydomonas reinhardtii* (Perron et al., 1999), and, among stramenopiles, in a diatom, *Toxarium undulatum* (Ruck et al., 2017), and most recently *D. speculum*, but not *R. marina*, *F. parvula*, *P. elastica* or other taxa (Han et al., 2019). Group II introns have also been reported in a handful of other plastid genes in other taxa (Perrineau et al., 2015). The intron within the diatom *psaA* gene lacks the En domain that confers mobility, and the *C. reinhardtii* chloroplast genome lacks this domain as well. In contrast, both the *D. speculum* and DEC-IX *psaA* genes contain a fully functional mobility element. The field transcriptomes mapping to this intronic region indicates its expression levels are similar to *psaA* (Figure 5A).

### Mixotrophy in Dictyochophytes

It is long known that some groups of phytoplankton engage in phagotrophy. These predatory mixotrophs prey upon other microbes and photosynthesize, an important factor because, depending on the extent of prey consumption and respiration versus photosynthesis, contributions to primary production are altered (Mitra et al., 2014; Flynn et al., 2019). Further, predatory mixotrophs are recognized as being quantitatively important in marine ecosystems (Worden et al., 2015; Ward and Follows, 2016; Edwards, 2019). Field experiments indicate that mixotrophs contribute close to 50% of total bacterivory at an oligotrophic site in the Mediterranean (Unrein et al., 2007) and in open ocean areas along a north-south transect in the Atlantic Ocean (Hartmann et al., 2012). The relative importance of predatory mixotrophs may vary with depth, for example a Sargasso Sea study found that 50% of photosynthetic nanoflagellates consumed prey in the surface mixed layer, while only 0.5% ingested prey in the DCM (Arenovski et al., 1995). Further, based on modeling studies, the combination of high light intensities and low availability of dissolved nutrients (as would occur in the surface) has been suggested to favor mixotrophs over photoautotrophic algae in oligotrophic surface waters (Duhamel et al., 2019; Edwards, 2019).

The phylogenetic affiliations of predatory mixotrophs remain poorly characterized, especially in offshore oligotrophic waters. Identification based on FISH showed high rates of bacterivory by haptophytes in the Mediterranean Sea, as well as cryptophytes and dinoflagellates (Unrein et al., 2014), the latter two being well recognized mixotrophic groups, including in oligotrophic ocean regions (Stoecker, 1999; Duhamel et al., 2019). By the same approach, haptophytes and chrysophytes (another stramenopile lineage) were found to feed on *Prochlorococcus* in the Atlantic subtropical gyres (Hartmann et al., 2013). Moreover, an obligate-predatory strategy has been reported for two cultured marine chrysophytes belonging to the *Ochromonas* genus, which, although actively photosynthesizing in the light, cannot achieve positive growth rates by photosynthesis alone, and one of which can live without light (Wilken et al., 2020).

Microscopy on field samples comes with its own set of challenges, including that FISH requires sequence information for the design of probes with specific targets. Our results provide target sequence information and an impetus to examine dictyochophytes in the field. This may help to fill in gaps, as in the Mediterranean study discussed above, where only about 50% of mixotrophic flagellates could be identified either morphologically or by the FISH-probes employed (Unrein et al., 2014). To date feeding by dictyochophytes has not been targeted in FISH studies, but situations in which they dominate amplicon reads from eukaryotic phytoplankton are ecologically similar to the oligotrophic surface waters of the Sargasso Sea (Arenovski et al., 1995), where a high proportion of actively feeding photosynthetic flagellates had been observed and the dominant phytoplankton are cyanobacteria (Figure 1C). In combination with our observation that flow sorted phytoplankton cells containing acid vacuoles are substantially composed of photosynthetic dictyochophytes, this suggests that the success of at least several dictyochophyte clades at BATS involves predatory mixotrophy - and that this is likely a means by which summertime photosynthetic eukaryotes acquire nutrients and can co-exist with cyanobacteria in the upper water column. The higher amplicon abundances of dictyochophytes relative to diatoms and other eukaryotic phytoplankton outside the picoplankton size class, may further reflect a capacity to outcompete these other eukaryotic taxa for nutrients by circumventing reliance on transporter affinity and an optimized surface to volume ratio.

Various lines of evidence indicate that predatory mixotrophy is at least common within dictyochophytes. Members of the Pedinellales from freshwater and marine environments have been reported as predatory mixotrophs (Sekiguchi et al., 2003), and photosynthetic freshwater *Pseudopedinella* can be important bacterivores in lakes (Gerea et al., 2016). The purely heterotrophic dictyochophyte species *Pteridomonas danica* and *Ciliophrys infusionum* lost photosynthetic capacity independently from each other, although both retain a non-photosynthetic plastid (Sekiguchi et al., 2002). DEC-X had an ill-supported position in the phylogenetic reconstructions and potentially could be a heterotrophic predator, since, although it retains a plastid 16S rRNA gene and presumably a plastid genome, it lacked chlorophyll fluorescence and was selected by having a stained vacuole (Supplementary Figure S7). In contrast to DEC-X, DEC-IX clearly retained a fully functional plastid, showing both chlorophyll fluorescence and expression of key photosynthetic genes. Evidence that a marine *Florenciella* species (Supplementary Figure S9; 98.5 – 99.8% nt identity to *F. parvula*) could be a predatory mixotroph comes solely from 18S rRNA data from an on-deck SIP experiment in the Pacific Ocean (Frias-Lopez et al., 2009) during which these taxa incorporated ^13^C from labeled cyanobacteria. Predatory mixotrophic trophic modes could thus be widely represented among diverse dictyochophytes, potentially including some of the environmental clades recovered here. The fact that 74 ± 15% of the dictyochophyte amplicons in our oligotrophic surface ocean samples do not have cultured counterparts calls for targeted efforts to characterize their physiology and ecological roles.

## Conclusion

Our field studies show that similar eukaryotic phytoplankton community structure patterns can be observed along different zones of the ENP when compared to seasonal periods in the Sargasso Sea. Prasinophyte algae comprise the bulk of eukaryotic amplicons in mesotrophic conditions and the deep mixing period at BATS, but among stramenopiles, pelagophytes are also notable under these conditions. We observed considerable diversity in pelagophytes and found that one species in particular, *P. calceolata*, persisted at the DCM in oligotrophic regimes. Additionally, a previously undescribed environmental clade, PEC-VIII, was also abundant among stramenopile amplicons. However, it was dictyochophytes that dominated eukaryotic phytoplankton amplicons in those regions of the water column where nutrient availability was at its lowest, regions where prior studies indicate mixotrophy is most prevalent. The cell sorting experiments indicated that the taxa comprising natural dictyochophyte populations have food vacuoles (Wilken et al., 2019), implying that they are predatory mixotrophs. Of note, both diatoms and at least some dictyochophytes require Si, opening the potential for competitive exclusion between these taxa, and for both to be agents of biomass export. We propose that dictyochophytes acquire nutrients from prey, circumventing direct competition with *Prochlorococcus* and *Synechococcus* for inorganic nutrients. This would enable them to contribute significantly to net primary production in the oligotrophic regions without relying on uptake of scarce dissolved nutrients.

Our findings support the outcomes of models that a synergistic coupling exists between prey ingestion for nutrients, and photosynthetic acquisition of carbon and energy, which enables mixotrophs to be successful in stable oligotrophic gyres (Duhamel et al., 2019; Edwards, 2019). Mixotrophic behavior also has consequences when assimilated into global food web models, shifting biomass to larger size classes and thereby enhancing sinking carbon flux (Ward and Follows, 2016). Thus, although the amount of carbon traversing food webs via mixotrophic inputs and the distribution of mixotrophic behaviors across algal diversity remain sparsely explored, there are mounting reasons to regard mixotrophy as a widespread behavior that significantly alters carbon cycling. Our results, combined with outcomes from modeling studies (Ward and Follows, 2016; Edwards, 2019), indicate that the dynamics of these dictyochophytes and other mixotrophs are likely to be sensitive to environmental change connected to enhanced ocean stratification, including changes in the duration and intensity of light exposure.

## Materials and Methods

### Oceanographic Sampling

BATS samples were collected between August 1991 to February 1994, and September 1997 to January 2004 as in Carlson et al. (2009). For ENP study sites, 15 samples were collected on four cruises, WFAD09, CANON11, C0912 and CN13ID, from 2009, 2011, 2012 and 2013, 8 sequenced for a prior cyanobacterial study (Sudek et al., 2015) and 7 sequenced herein (Supplementary Table S5). Samples were collected using Niskin bottles mounted on a rosette equipped with an SBE9 conductivity, temperature and depth (CTD) sensor (Sea-Bird Electronics). Samples for nucleic acid extraction were collected by filtering 500–2000 ml seawater through 0.2 μm pore size polyethersulfone membrane filters (Supor 200, Pall Gelman). Filters were placed into sterile cryovials, flash-frozen in liquid nitrogen and transferred to −80°C until further use. ENP RNA samples were collected by filtering 15–20 L of seawater onto a 3 μm pore-size, 142 mm diameter Versapor filter (Pall Corporation), storing at −80°C and extracting as described previously (Needham et al., 2019). These came from five 2014 samples, two previously sequenced (Needham et al., 2019). Others were processed and sequenced herein, specifically from 20 March at 10 m (ERS3865411) and 20 m (ERS2592094), 5 May at 10 m (ERS3865412) and 20 m (ERS3865413) and 2 April at 20 m (ERS2592093). Samples from 2 April 2014 and 5 May 2014 were pre-filtered through a 30 μm mesh, while those from 20 Mar 2014 were not. In 2013, additional metatranscriptome samples were collected at 10 m at 67–70 and 100 m at Station 67-155 and pre-filtered through a 20 μm mesh before final filtration, freezing and processing. Nutrient data were acquired as described previously for BATS (Treusch et al., 2009) and the ENP (Sudek et al., 2015).

### DNA Extraction

Briefly, 384 monthly BATS samples (from August 1991 to February 1994 and September 1997 to January 2004) were extracted as described previously in Treusch et al. (2009). The 15 ENP samples were extracted using the DNeasy Plant Mini Kit (Qiagen) with a modification of the manufacturer’s protocol, as described previously (Demir-Hilton et al., 2011).

*Pelagomonas calceolata* CCMP1756 was acquired from the National Center for Marine Algae and Microbiota (NCMA) and grown on L1-Si medium at 21°C under a 14:10 h light:dark cycle with 100 μmol quanta m^–2^ s^–1^. The culture was harvested during exponential growth by centrifugation at 8,000 × *g* for 10 min. Most of the supernatant was removed, the pellet resuspended in the remaining ∼1 ml supernatant, transferred to a microcentrifuge tube and pelleted again. DNA was extracted from pellets using the DNeasy Plant Mini Kit following the manufacturer’s protocol.

### 16S rRNA Gene V1-V2 Amplicon PCR and Sequencing

BATS amplicons were generated as part of a prior study and ENP sequencing used the same 16S rRNA V1-V2 primers 27FB (5′-AGRGTTYGATYMTGGCTCAG-3′) and 338RPL (5′-GCWGCCWCCCGTAGGWGT-3′) as in Vergin et al. (2013), Sudek et al. (2015) using the 454 platform. Twelve samples per plate were sequenced using Roche/454 GS FLX Titanium platform (Roche 454 Life Sciences). Quality control (QC) of 454-pyrosequenced reads was performed using published methods (Hamady et al., 2008). This amounted to 907,022 ENP amplicons in total after QC, as well as 2,540,966 16S V1-V2 amplicons after QC from 384 BATS samples (latitude ranges from 31.164 to 31.906 and longitude from −64.679 to −63.773).

### Reference Alignments and Trees for Amplicon Placement

16S rRNA V1-V2 amplicons were initially parsed using PhyloAssigner v6.166 (Vergin et al., 2013) with amplicons from either plastids or cyanobacteria first taxonomically placed using global cyanobacterial and plastid phylogenetic trees, as described in Sudek et al. (2015), Choi et al. (2017). Briefly, this method aligns amplicons to the unmasked alignment from the phylogenetic reconstruction based on full length sequences and requires statistical support for the placement to be considered valid (Matsen et al., 2010). Here, in addition to using the previously published PhyloAssigner alignments (Choi et al., 2017), we took a total of 270 high-quality, near full-length 16S rRNA gene sequences from 84 described stramenopile species and 182 environmental sequences from undescribed stramenopile taxa, including many from 2007 ENP samples (Choi et al., 2017), and aligned them using MAFFT v7.055b (Katoh and Standley, 2013) with default parameters. After manual curation this alignment was used to generate a high-quality stramenopile reference tree, which included four rhodophyte sequences as an outgroup. Positions with gaps were masked using Gblocks v0.91b (Castresana, 2000) and phylogenetic inferences were done using Maximum Likelihood inference implemented in RAxML v8.0.0 (Stamatakis, 2014) under the gamma corrected GTR model of evolution with 1,000 bootstrap replicates (-m GTRGAMMA -f a -# 1,000 parameters) based on the analysis of 1,033 homologous positions. Another maximum likelihood inference was made using PhyML v3.0.1 (Guindon et al., 2010) with the substitution model of gamma corrected GTR and 100 bootstrap replicates (-m GTR -f e -v e -c 8 -a e -b 100 -BEST -n_rand_starts 10). Additional phylogenetic reconstructions were performed in MrBayes v3.2.6 (Ronquist et al., 2012) with the parameters of lset nst = 6 rates = invgamma ncat = 6, and ngenval = 10,000,000 samplefreqval = 1,000 and tempval = 0.200, and the final tree figure was produced with assistance from FigTree v1.4.3 and topology from RAxML (Supplementary Figure S2). More refined specific reference alignments and phylogenetic trees were constructed for two stramenopile classes, pelagophytes and dictyochophytes (Figure 2B and Supplementary Figure S5) using the same approaches described above. Briefly, a total of 68 and 61 sequences were used for pelagophytes and dictyochophytes with 1,210 and 1,066 homologous positions, respectively. To improve the resolution of the trees, 16S rRNA gene reference sequences from the MMETSP datasets (10 for pelagophytes, 5 for dictyochophytes) were added.

In order to ascertain the accuracy of placement of amplicon sequences on the unmasked alignment by PhyloAssigner we performed quality control with a set of known sequences. Specifically, for both the pelagophyte and dictyochophyte reference trees, 10 sets of V1-V2 sequences were generated from 50 near-full length 16S rRNA gene sequences and tested by running them through PhyloAssigner, with the new reference trees, to verify correct classification. The placement results were 100% correct.

### Data Set Description and Statistical Analysis

For BATS, 384 monthly samples collected over a 12 year period from 1991 to 2004 were aligned using the month of deepest mixing (month “0”) as the reference point to establish months −1 to +10 (Carlson et al., 2009). Because our focus was on eukaryotic phytoplankton, samples with low amplicon sequencing depth (and poor statistical quality for high taxonomic resolution plastid analysis) were excluded, resulting in retention of 191 samples having > 1,000 total 16S V1-V2 rRNA amplicons and > 100 representing plastids. Of these, 77 were from the surface (0–10 m, 8,025 ± 4,896 16S amplicons sample^–1^) and 27 from the SCM/DCM (40 – 120 m, 7,395 ± 3,528 16S amplicons sample^–1^). Eighty-seven came from other depths between 40 and 300 m (7,612 ± 4,592 16S amplicons sample^–1^). For stramenopile analyses samples were excluded with < 100 for total stramenopile, or < 20 for an individual stramenopile class, amplicons.

Within samples, relative abundance for each phylogenetic subgroup was calculated based on the total number of amplicons from the respective higher taxonomic group (Supplementary Figure S12). Additionally, because *Prochlorococcus* and *Synechococcus* generally have one and two copies of the rRNA operon, respectively, the latter were divided by two prior to computing percentages as in (Sudek et al., 2015). At each depth averages and standard deviations (SD) of phylogenetic group abundances were computed per month (−1 to +10) at BATS (Carlson et al., 2009). Due to uneven sample numbers for each month (data S1), the unweighted mean, notated here as μ x̄, equation (1), was calculated as

(1) $$\mu_{\bar{x}}=\frac{\sum_{i}\mu_{{\bar{x}}_{i}}}{n}$$

and the pooled SD, notated here as σ x̄, equation (2), was calculated as

(2) $$\sigma_{\bar{x}}=\sqrt{\frac{\sum_{i}\sigma_{{\bar{x}}_{i}}^{2}}{n}+\frac{\sum_{i<j}{\left(\mu_{x_{i}}-\mu_{x_{j}}\right)}^{2}}{n^{2}}}$$

where *n* is the number of means. Due to limited SCM/DCM data, averages and SDs at this depth were computed using monthly data with ≥ 3 samples that met amplicon cutoff criteria (months 0, DM; +4, +6 and +9 stratified).

Fifteen ENP samples were analyzed using the same PhyloAssigner approach. Overall ENP sequencing depth was greater, with on average 11,300 ± 6,894 total and 3,366 ± 2,641 plastid-derived amplicons recovered per sample excluding one sample with 63,526 total and 26,672 plastid-derived amplicons.

### Hierarchical Clustering

Hierarchical clustering was carried out using log-transformed normalized unweighted means of relative amplicon abundances. The approximately unbiased *p*-values (%) as well as bootstrap probabilities were computed via multiscale bootstrap resampling with 10,000 replications using the R package Pvclust (Suzuki and Shimodaira, 2006), modified to allow Bray-Curtis similarities for distance calculations.

### qPCR Primer Design, Testing and Implementation

To generate 18S rRNA gene insert-bearing plasmid standards for qPCR, the 18S rRNA gene was amplified from *P. calceolata* CCMP1756 using the primers 5′-ACCTGGTT GATCCTGCCAG-3′ and 5′-TGATCCTTCYGCAGGTTCAC-3′ (Moon-Van Der Staay et al., 2001). PCR cloning and plasmids purification was performed as in (Demir-Hilton et al., 2011). Clones were bidirectionally (Sanger) sequenced using Big Dye Terminator chemistry (Applied Biosystems) with plasmid primers M13F/M13R and the two internal primers 502f and 1174r (Worden, 2006).

To quantify *P. calceolata* abundance, a *P. calceolata*-specific TaqMan primer-probe set was developed (Supplementary Table S6) after retrieving cultured pelagophyte and environmental 18S rRNA gene sequences from GenBank. Primers specific to *P. calceolata* were designed manually and a *P. calceolata-*specific probe sequence was identified using Beacon Designer 8.14 (PREMIER Biosoft International). Melting temperature and secondary structures were checked using OligoAnalyzer 3.1 from IDT SciTools. The primer-probe set specificity for *P. calceolata* was validated using *Pelagococcus subviridis* and *Aureococcus anophagefferens* (Supplementary Table S7). qPCR including inhibition tests and analysis was performed as described previously (Demir-Hilton et al., 2011) for fourteen environmental samples from mesotrophic and oligotrophic ENP sites that were also amplicon sequenced.

### Single-Cell Sorting

For flow sorting, ENP seawater was collected on 2 April 2014 (20 m) at Station M1 and 5 May 2014 (10 m) at M1 and M2. Seawater was run through a BD Influx flow cytometer with two lasers (488 nm and 355 nm excitation), operated using sterile nuclease-free 1 × PBS as sheath (Cuvelier et al., 2010). Prior to sorting, samples were pre-filtered through a 30 μm nylon mesh and concentrated 30–50 times by gravity filtering over a 0.8 μm Supor (Pall Gelman). Two stains, LysoTracker Green DND-26 (25 nM, final concentration) and LysoSensor Blue DND-167 (1 μM, final concentration) with emission collected through a 520/35 nm bandpass and a 435/30 nm bandpass, respectively, natural Forward Angle Light Scatter (FALS, a proxy for cell size) and chlorophyll fluorescence (692/40 nm bandpass) were used in a variety of gating scenarios. Stained samples were incubated for 10 to 20 min before being run. Unstained controls were run to discern positive signals from stains. 384-well plates were illuminated with UV for 2 min prior to performing the sorts and selected cells were then sorted using the Single-cell sorting mode from the BD FACS Sortware (software v1.0.0.650), ensuring that only one cell would be sorted into each well. The drop delay and sort quality were controlled by sorting known numbers of fluorescent beads and counting on a microscope. Negative and positive controls on each plate involved leaving a subset of wells empty and having a column of wells receive 20 cells, respectively. Plates were covered with foil and immediately frozen at −80°C.

### Single-Cell Genome Amplification and Analysis

Whole genome amplification (Cuvelier et al., 2010) and rRNA amplicon sequence analysis of sorted dictyochophyte cells followed the methods detailed previously (Needham et al., 2019) with the latter using pooled triplicate reactions from the Illumina adapted TAReuk454FWD1/TAReukREV3 primers for the 18S rRNA V4 region (Stoeck et al., 2010). Sequences were *de novo* clustered at 99% nt identity by UCLUST. The dictyochophyte wells had only a single 18S amplicon type. Whole genome sequencing of two single cells was then performed via Illumina HiSeq (Illumina), generating 14,848,205 and 15,058,189 PE 2 × 251 bp reads. Analysis of genome sequences from the dictyochophyte cells were quality controlled as previously detailed (Needham et al., 2019). For assembly of the uncultured dictyochophyte, paired and unpaired quality filtered reads from the two dictyochophyte-containing wells were initially assembled as individual single cell assemblies as well as a co-assembly of both samples using SPADES v 3.11.0 (Bankevich et al., 2012) as previously described (Cuvelier et al., 2010). Then, mapping of reads to the assembled contigs and identification of chloroplast genomes was performed in Anvi’o v3 as previously described (Eren et al., 2015), and enabled by identification of chloroplast rRNA genes with metaxa2 (Bengtsson-Palme et al., 2015). The chloroplast contigs from the two wells were determined to have > 99.9% nt identity to one another via the *ani.rb* function within the enveomics package (Rodriguez-R and Konstantinidis, 2016). A single well was chosen as a representative of the dictyochophyte chloroplast genome assembly and used for the downstream analysis of metatranscriptome mapping. The complete chloroplast genome was annotated using DOGMA (Wyman et al., 2004) and all intergenic spaces were manually scanned using BLAST for ORFs or additional proteins that might not be present in chloroplast genomes from other phytoplankton.

### RNA Extraction and Sequencing

RNA was extracted using the TotallyRNA kit (Life Technologies) with the following initial steps to maximize lysis and accommodate the large filter: in a sterile petri dish 2 ml of lysis buffer from the kit was added to the frozen filter half. The filter was then cut into six pieces and the filter pieces along with buffer transferred into two 2 ml screw cap tubes pre-filled with ∼200 μl of a 1:1 mixture by volume of 0.1 mm and 0.5 mm diameter autoclaved glass beads (Biospec Products). After one minute of bead beating filter pieces and lysis buffer from the two tubes were recombined in a 15 ml screw cap tube and a further 3 ml lysis buffer added. The remainder of the extraction followed the manufacturer’s instructions. DNA was digested using the TurboDNA-free kit (Life Technologies) following manufacturer’s instructions. RNA integrity was evaluated on a Bioanalyzer (Agilent) and quantity determined on a Qubit fluorometer (Life Technologies). RNA yield ranged from 7.1 to 19.5 μg and after library construction was sequenced via Illumina HiSeq. Quality-filtered metatranscriptome reads (QC performed as above) were mapped to the uncultured dictyochophyte chloroplast genome assembly and *P. calceolata* chloroplast genome (accession JX297813) via bbmap.sh (v37.17) (Bushnell, 2014) at a sequence similarity cutoff of 0.99. Mapped reads were parsed via HTSeq-count (Anders et al., 2015), binned at 100 bp increments and placed on the circos (Krzywinski et al., 2009) plot based on the start position of their match in the uncultured dictyochophyte plastid genome.

## Data Availability Statement

The datasets generated for this study can be found in GenBank under accession numbers: MN275232, SRR7789640–SRR7789654, and ERS3865411–ERS3865415.

## Author Contributions

AZW and SJG designed the study. AZW designed ENP cruises in collaboration with FPC. VJ collected ENP samples, with contributions from SW and SS. SS and VJ performed amplicon sequencing on ENP samples. CJC performed phylogenetic analyses, with input from CB and HA. CJC analyzed amplicon data. CP and CB performed single-cell sorting and initial analyses. CJC and DMN performed metagenomics and transcriptomic analyses. CJC, SJG, and AZW wrote the manuscript with edits from all authors, especially SW and HA.

## Conflict of Interest

The authors declare that the research was conducted in the absence of any commercial or financial relationships that could be construed as a potential conflict of interest.
