# Supplementary material for: Seasonal and Geographical Transitions in Eukaryotic Phytoplankton Community Structure in the Atlantic and Pacific Oceans
Source: Front Microbiol. 2020 Sep 30;11:542372. doi: 10.3389/fmicb.2020.542372 (PMC7554337; doi:10.3389/fmicb.2020.542372)

Supplementary Material

Seasonal and geographical transitions in eukaryotic phytoplankton community structure in the Atlantic and Pacific Oceans

Chang Jae Choi, Valeria Jimenez, David M. Needham, Camille Poirier, Charles Bachy, Harriet Alexander, Susanne Wilken, Francisco P. Chavez, Sebastian Sudek, Stephen J. Giovannoni, and Alexandra Z. Worden^*^

*** Correspondence:**Alexandra Z. Worden
azworden@geomar.de

**This PDF file includes:**

Supplementary Figures S1-S12

Supplementary Tables S1-S9

**Other Supplementary Materials for this manuscript includes the following:**

Supplementary Data S1-S3 (Microsoft Excel format). Detailed amplicon numbers (Supplementary Data S1), Dictyochophyte chloroplast genome features (Supplementary Data S2) and Metatranscriptome mapping (Supplementary Data S3) analyzed in this study.

**TABLE OF CONTENTS**

**SUPPLEMENTARY FIGURES (pp 3-17)**

1. Phytoplankton distribution in the SCM/DCM at BATS p 3
2. Phylogenetic reference tree of photosynthetic stramenopiles p 5
3. Hierarchical clustering of strameopile amplicons at BATS surface waters p 6
4. Depth distribution of stramenopile amplicons at BATS p 7
5. High-resolution 16S rRNA gene phylogeny of dictyochophytes p 9
6. Phytoplankton distribution from Line-67 in the ENP p 10
7. Single-cell sorting of wild uncultivated dictyochophytes p 11
8. Modified dictyochophyte phylogeny based on full length plastid 16S p 13
9. Phylogeny of dictyochophytes based on full length 18S rRNA genes p 14
10. Phytoplankton distribution from Line-67 coastal waters in the ENP p 15
11. Hierarchical clustering of stramenopile ampliconss from BATS and ENP p 16
12. Overall description of 16S rRNA gene V1-V2 amplicon analysis pipeline p 17

**SUPPLEMENTARY TABLES (pp 18-26)**

1. The relative abundance of overall phytoplankton amplicons p 18
2. The relative abundance of eukaryotic phytoplankton amplicons p 19
3. The relative abundance of photosynthetic stramenopile amplicons p 20
4. Pelagophyte clade distribution in relation to depth at BATS p 21
5. Physico-chemical parameters attained for the analyzed amplicon samples p 22
6. *P. calceola* specific primer-probe details for qPCR analysis p 23
7. Specificity validation of *P. calceolata* specific primer-probe set p 24
8. Accessions for key thiamin biosynthesis genes from dictyochophytes p 25
9. Accessions and sample details p 26

**Supplementary Figures**

**
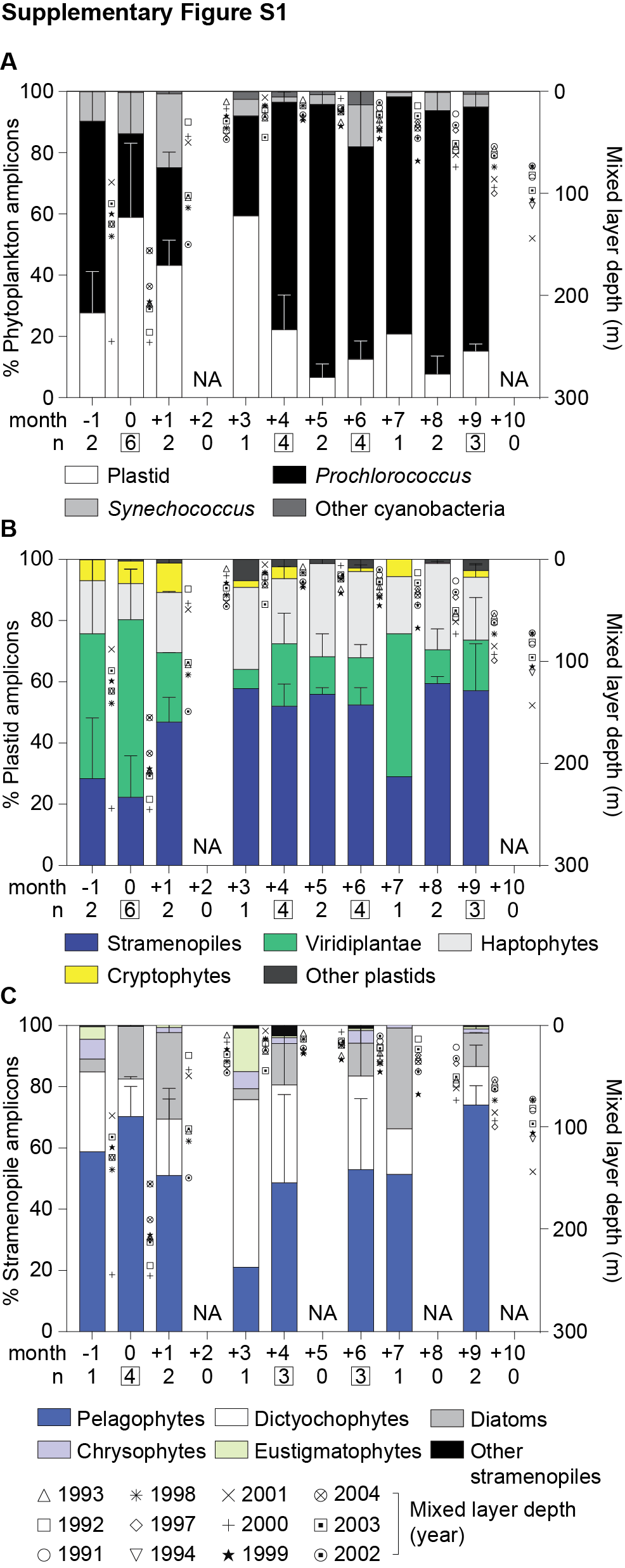
**

**Supplementary Figure S1** Phytoplankton distribution in the northwestern Sargasso Sea (BATS) at depth (SCM/DCM). **(A)** The relative abundance of overall phytoplankton 16S V1-V2 amplicons expressed as a percentage of amplicons phylogenetically assigned to plastids, *Prochlorococcus*, *Synechococcus* and other cyanobacterial groups, **(B)** major eukaryotic phytoplankton lineages and **(C)** photosynthetic stramenopiles illustrate broad spatiotemporal dynamics of phytoplankton diversity and distribution at depth (SCM/DCM). Monthly data from 1991 to 2004 at the BATS site are integrated over a 1 year time frame after adjusting to the month of the maximum mixed layer depth (month 0). Error bars represent the standard deviation coming from varied sample numbers, with the respective number being indicated by the ‘n’ indicated on the figures. Boxed n values indicate the number of samples used for developing unweighted means and pooled standard deviations (requirement of ≥ three samples at that sampling month for inclusion). Note that during DM nitrate was 0.191 ± 0.216 μM NO_3_^-^ (average of 7 samples out of 14 samples that were above the detection limit). In surface stratified waters nitrate averaged 0.096 ± 0.075 μM (5 measurements; with 72 others being below detection). Phosphate was below detection during DM (14 measurements attempted in total) and averaged 0.061 ± 0.027 μM when detectable (n=10, 67 below detection) in the surface stratified waters.

**
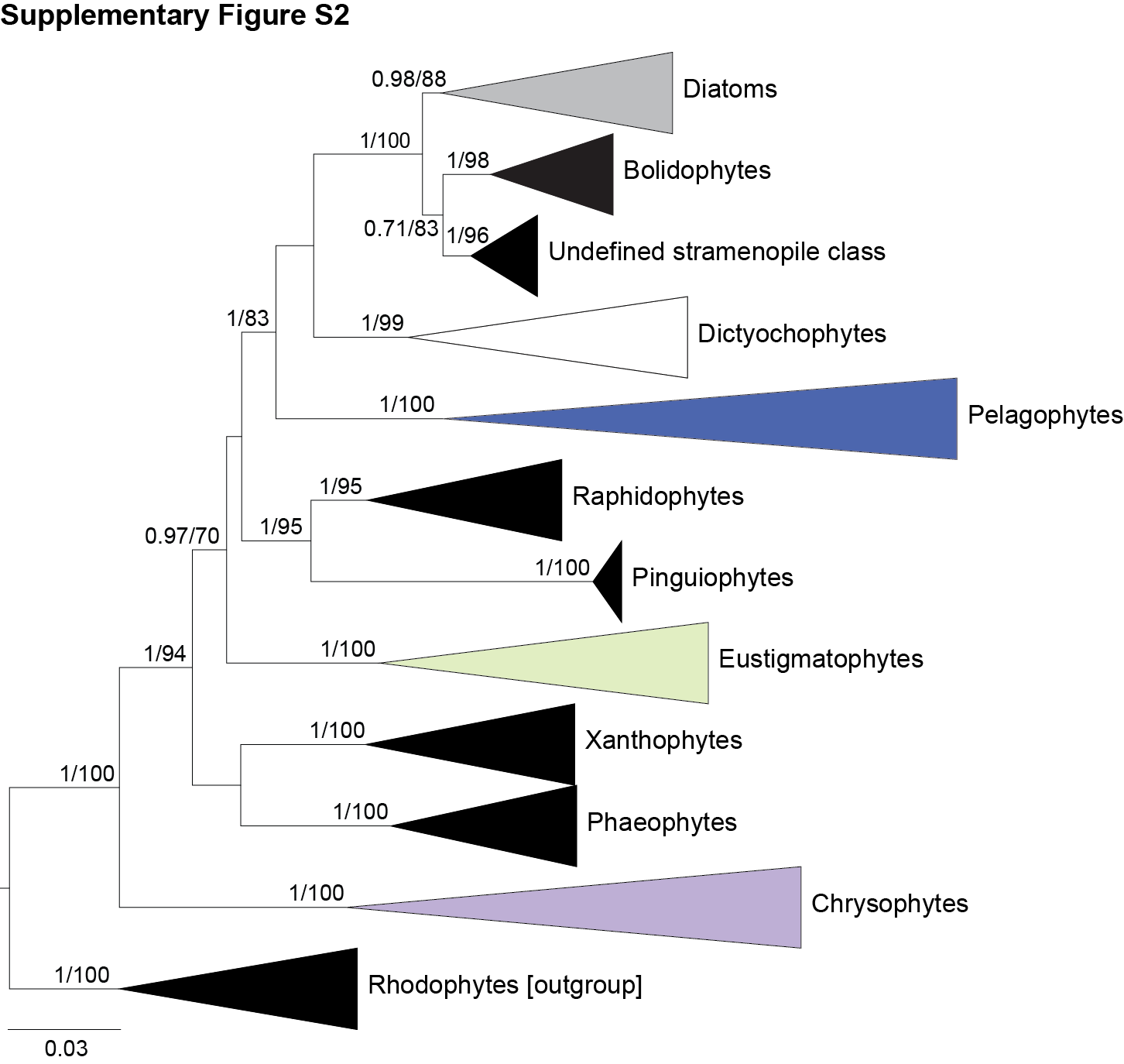
**

**Supplementary Figure S2** Phylogenetic reference tree used for classification of photosynthetic stramenopiles. The tree was constructed with Maximum Likelihood inference under the gamma corrected GTR model of evolution with 1,000 bootstrap replicates using 270 sequences (84 described, 182 undescribed stramenopiles, and 4 sequences from rhodophytes as an outgroup). Additional statistical node supports are indicated based on the Bayesian posterior probabilities. All 10 known photosynthetic stramenopile classes retained bootstrap support. One additional clade of uncultured sequences was identified that sistered the bolidophytes in a supported position, potentially representing an unrecognized class.


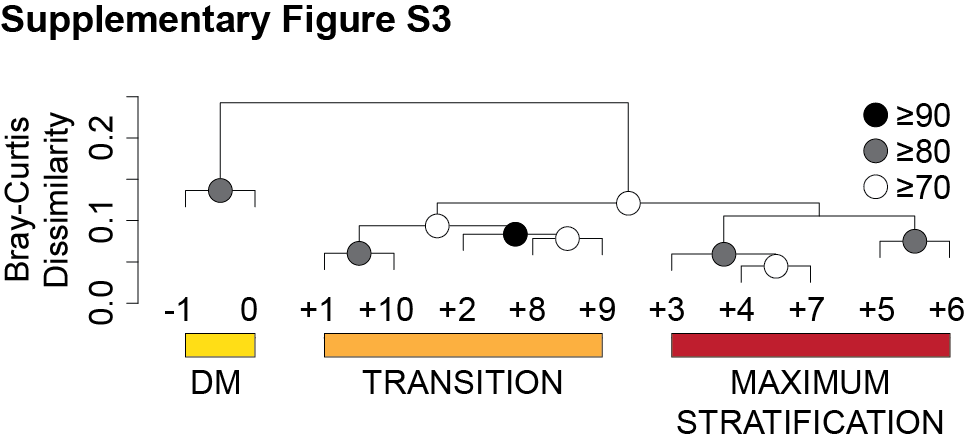


**Supplementary Figure S3** Hierarchical clustering analysis with Bray-Curtis dissimilarity based on the relative abundance of stramenopile amplicons in surface waters at BATS shows a distinct community shift along the seasonal gradients (DM, Transition and Maximum Stratification). Approximately unbiased (AU) probability values based on the multiscale bootstrap resampling (10,000 replications) were calculated and expressed as *p*-values (%).


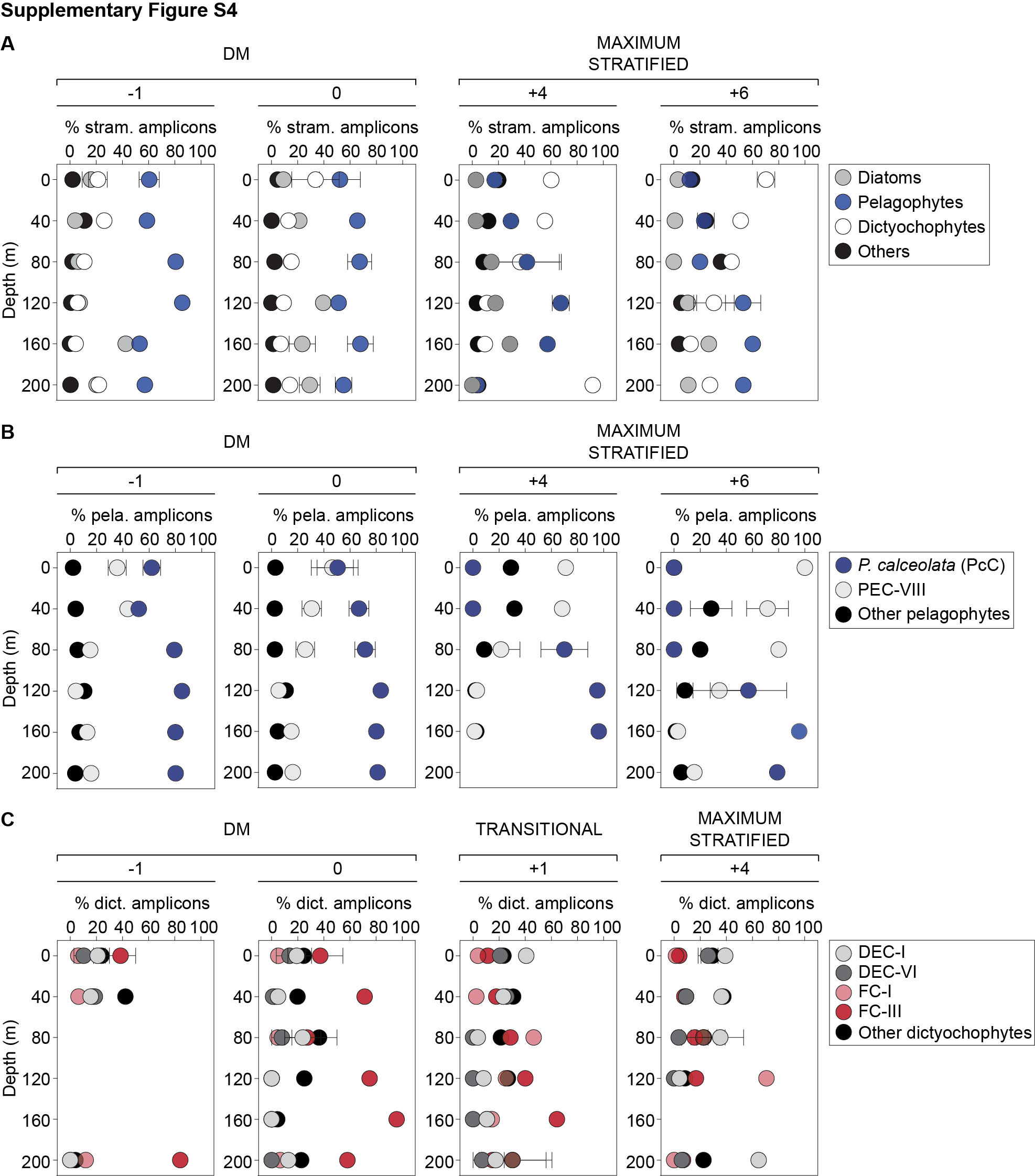


**Supplementary Figure S4** Relative stramenopile depth distributions during the months of deep mixing (DM) and Transitional and Maximum Stratified periods at BATS. These analyses are based on four months (indicated above each plot; varying depending on group analyzed) for which sufficient amplicon data was available. **(A)** Three major stramenopile classes, diatoms, pelagophytes and dictyochophytes and the combined minor classes are shown and represented as relative abundance of total stramenopile amplicons (See Supplementary Table S4). Note, amplicons from pelagophytes, dictyochophytes and diatoms represented 61 ± 16%, 16 ± 15% and 21 ± 13% of the total stramenopile amplicons, respectively, and other stramenopiles represented 2 ± 3% throughout the water column during DM months. During DM, higher contributions from diatoms at depth (120-200 m) relative to the surface (0 to 80 m) were observed (*p*<0.05, two-tailed Mann-Whitney *U*-test). **(B)** Pelagophyte relative distributions for the two most prominent clades, *P. calceolata* Clade (PcC) and Pelagophyte Environmental Clade VIII (PEC-VIII) with more minor clades grouped as other pelagophytes are shown and represented by the relative abundance of amplicons within pelagophytes. **(C)** Dictyochophyte clade depth relative distributions for clades for which sufficient amplicons were detected were analyzed in these depth profiles, i.e., DEC-I, DEC-VI, FC-I, FC-III, while those with lesser relative abundances were grouped as “others”. Error bars represent the standard deviation of the mean.

**
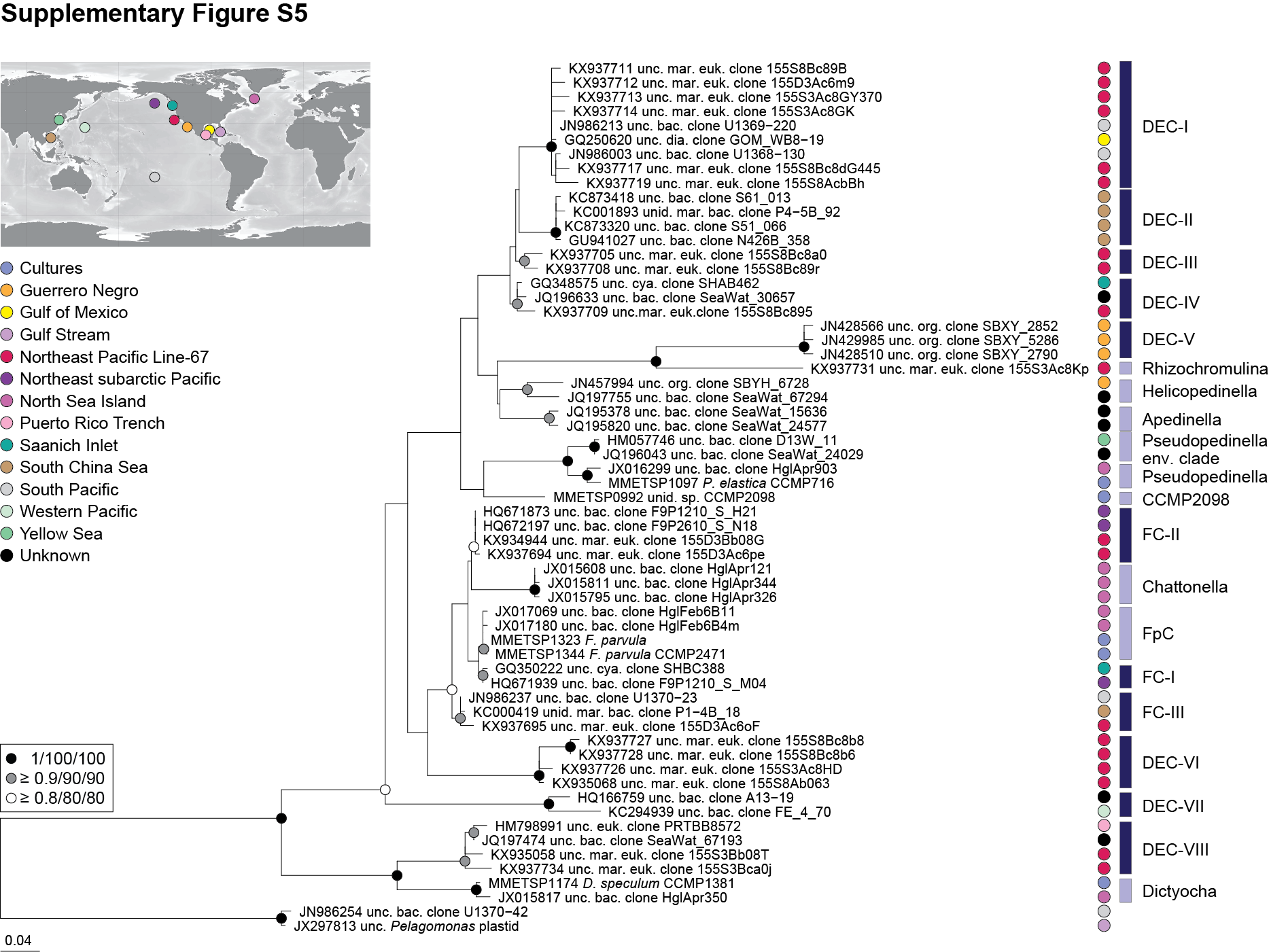
**

**Supplementary Figure S5** High-resolution 16S rRNA gene phylogenetic reconstruction of dictyochophytes, containing five sequences from cultures and 54 from environmental clones. This phylogenetic reconstruction using Maximum Likelihood inference under the gamma corrected GTR model of evolution with 1,000 bootstrap replicates delineated 20 clades, 11 of which have not been recognized previously, Dictyochophyte Environmental Clades I-VIII (DEC-I to DEC-VIII) and *Florenciella* Clade I-III (FC-I to FC-III). Note that in some cases only partial 16S sequences are available for a known culture, however with a 100% nt identity to an environmental sequence. In these cases we named the group for the culture (e.g. *Chattonella*), despite there being no full-length 16S sequence from a culture. Additional node statistical supports are indicated based on Bayesian posterior probabilities and another Maximum Likelihood inference (PhyML). Two pelagophyte sequences were used as an outgroup. Colors indicate oceanic region of origin.


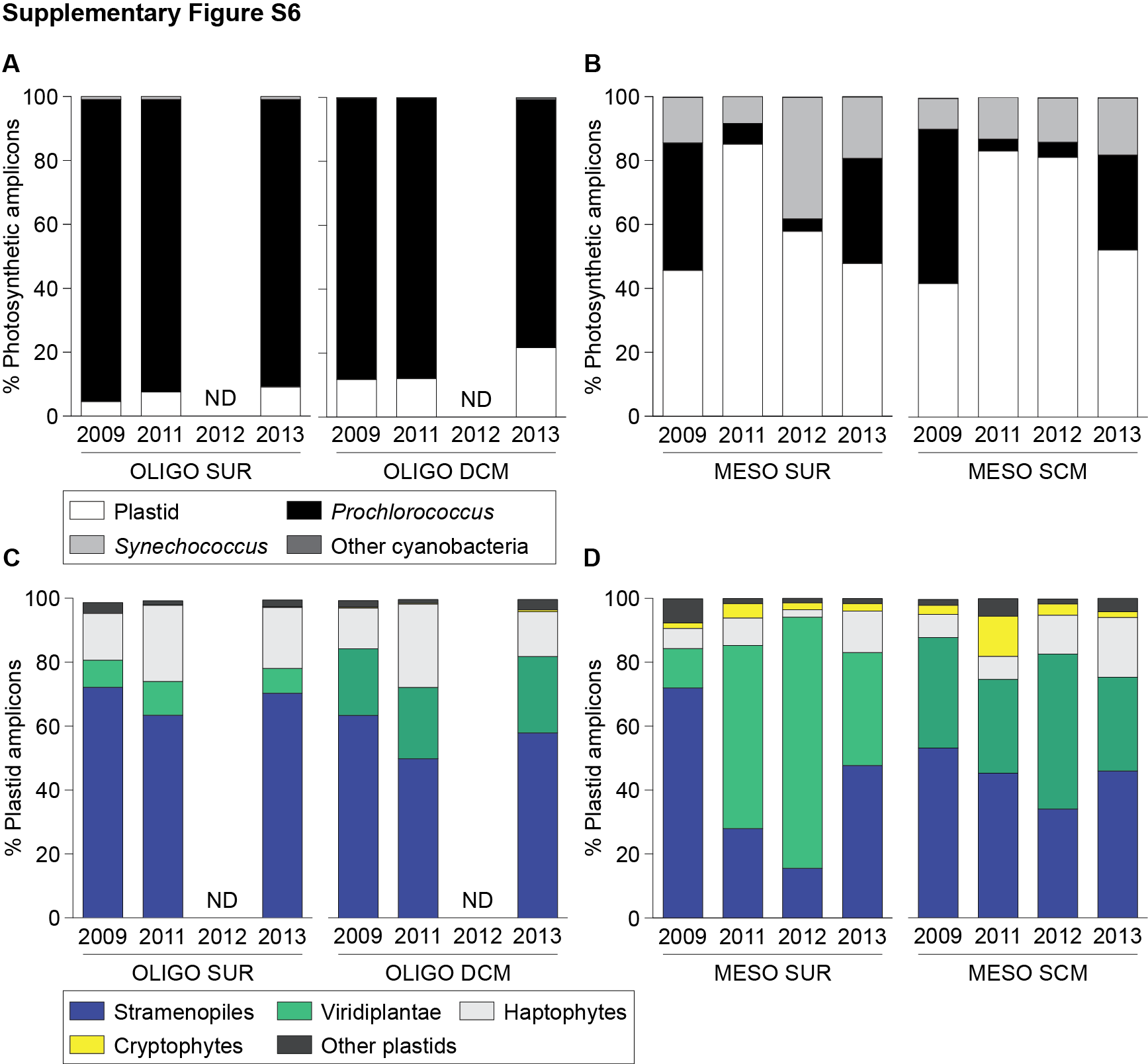


**Supplementary Figure S6** Phytoplankton diversity and distribution at the surface (SUR) and depth (SCM/DCM) on Line-67 in the ENP. The relative abundance of overall phytoplankton 16S V1-V2 amplicons expressed as a percentage of amplicons phylogenetically assigned to plastids, *Prochlorococcus*, *Synechococcus* and other cyanobacterial groups from **(A)** oligotrophic (OLIGO) and **(B)** mesotrophic (MESO) waters and major eukaryotic phytoplankton lineages from **(C)** oligotrophic (OLIGO) and **(D)** mesotrophic (MESO) waters illustrate broad spatiotemporal dynamics of phytoplankton diversity and distribution. Two trophic gradients, mesotrophic (MESO) and oligotrophic (OLIGO) waters from 2009 to 2013 are shown based on the four representative stations (Stations 67-60drift and 67-70, mesotrophic sites; Stations 67-135 and 67-155, oligotrophic open ocean sites). ND: no data. Surface ENP samples were obtained from 2 to 14 m depth and SCM/DCM samples at the chl *a* fluorescence maxima (31 to 41 m for SCM; and 81 to 105 m for DCM).

**
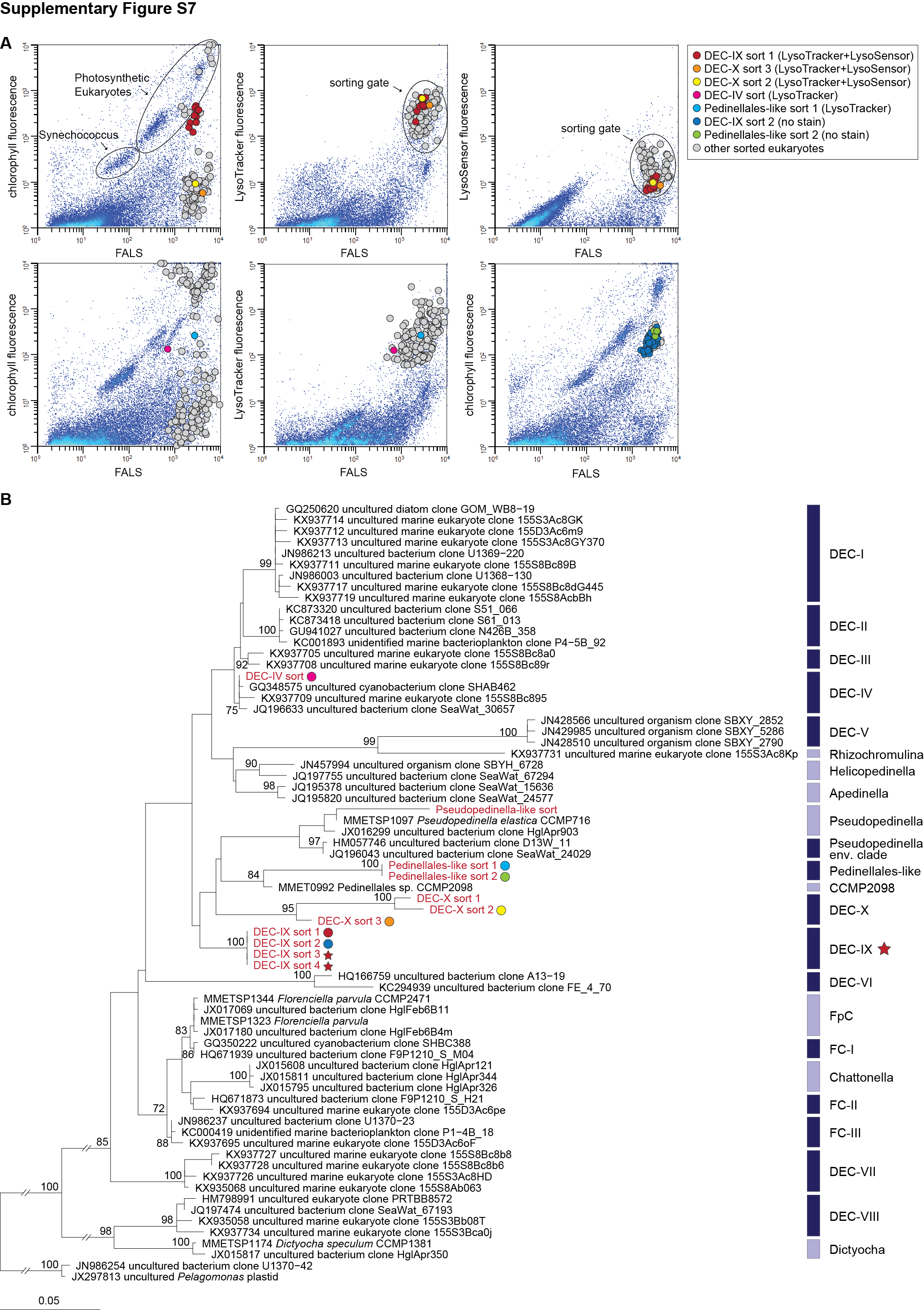
**

**Supplementary Figure S7** Single-cell sorting of wild uncultivated dictyochophytes. **(A)** Example two parameter histograms from sort experiment at Station M2 on 5 May 2014, with FALS (indicator of cell size) versus red (chlorophyll-derived) fluorescence (top/bottom left and bottom right panels), versus LysoTracker green fluorescence (top/bottom middle panels) and LysoSensor blue fluorescence (top right panel). The voltage for chlorophyll detection was very high, such that non-chlorophyll fluorescing cells are pulled on scale. Sort gate settings were based on all of these parameters and, as each cell was sorted, it was indexed for well position connected to listmode data. **(B)** Phylogenetic reconstruction of dictyochophytes based on the near full length 16S rRNA reference tree gene and amplicons from sorts (253 nt), analyzed using a setting to accommodate missing data (the V4 amplicons being much shorter than near full length) and also using PhyloAssigner. The chloroplast genome was sequenced from the DEC-IX Clade (red star). Note two of the sorted-cells were selected based on FALS and chlorophyll fluorescence without stains having been applied to the sample. Hence, for these two it was initially unclear whether a food vacuole is present or not. One of these was identical to another sort stained with LysoTracker+LysoSensor (DEC-IX) and the other was identical to Pedinellales-like sort 1 (which was stained with LysoTracker). Pedinellales-like sort 1 and 2 form a supported clade withPedinellales isolate CCMP2098 (96% nt identity)., Pedinellales-like sort 1 and 2 are mixotrophs. Pedinellales-like sort 1 and 2 are not identical to CCMP2098-like sort 1 in the 18S rRNA reference tree (Supplementary Figure S9). Colored circles correspond to the colored circles in the two parameter histograms in (A). The Maximum Likelihood tree was inferred by RAxML under the gamma corrected GTR model of evolution and was constructed based on the 1,066 positions, and for amplicons use of missing positions. Node supports are nonparametric bootstrap percentages obtained from two different Maximum Likelihood inferences, 1,000 replicates for RAxML and 100 replicates for PhyML. Additional node support indicated is based on the Bayesian posterior probabilities. Long branch lengths for the amplicon sequences from several sorted cells are likely artifacts, and compromise the result. This is an unavoidable consequence of using sequences with extensive “missing characters” (due to short length).


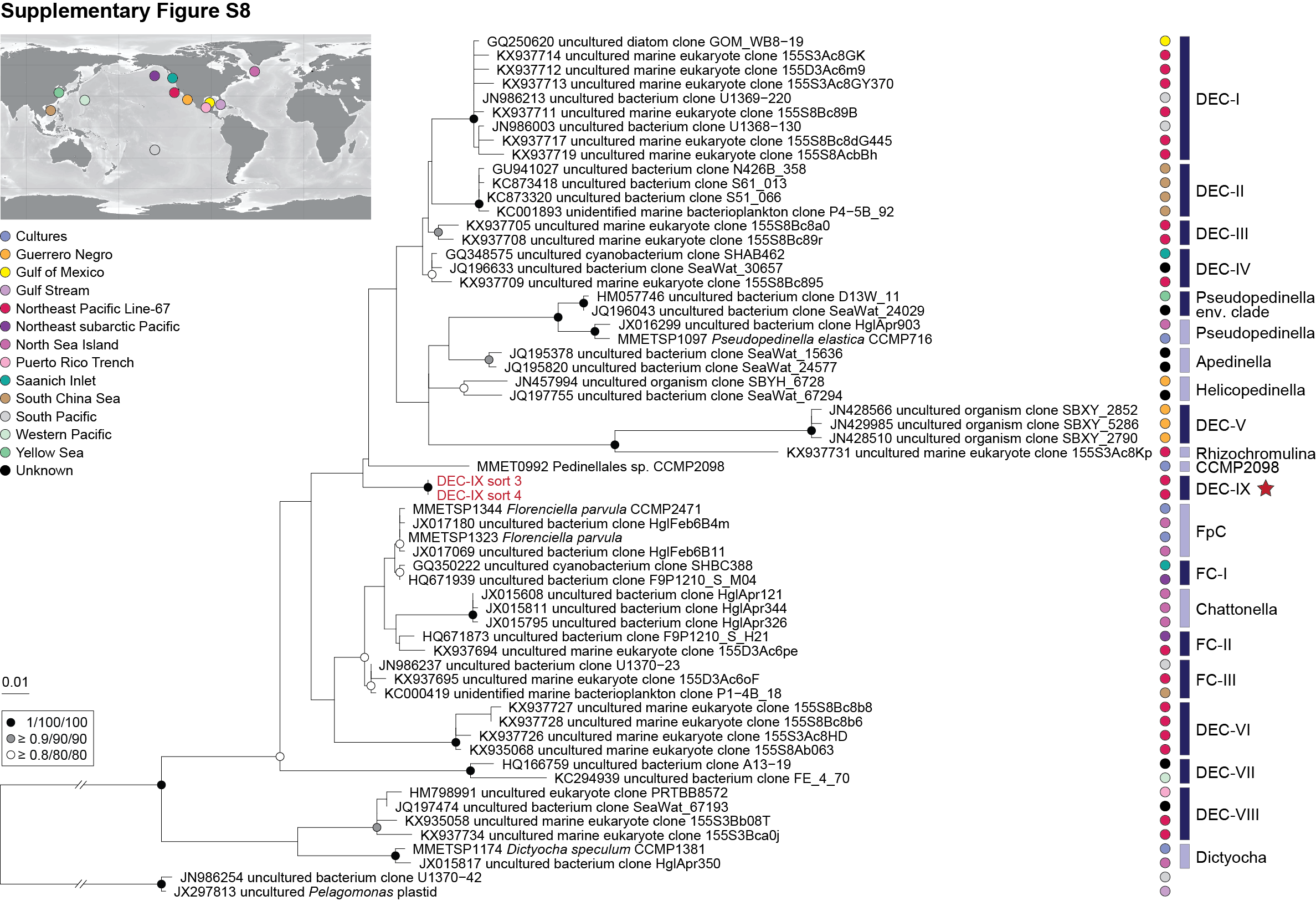


**Supplementary Figure S8** Modified phylogeny of dictyochophytes based on near full length plastid 16S rRNA genes (Supplementary Figure S5) for placement of sorted DEC-IX cells. The two sorted two cells with a star (red) were sequenced to near full length (1,401 nt). Maximum Likelihood tree inferred by RAxML under gamma corrected GTR model of evolution was constructed based on the 1,066 positions. Node supports are nonparametric bootstrap percentages obtained from two different Maximum Likelihood inferences, 1,000 replicates for RAxML and 100 replicates for PhyML. Additional node statistical support was indicated based on the Bayesian posterior probabilities. Colors indicate oceanic region of origin.


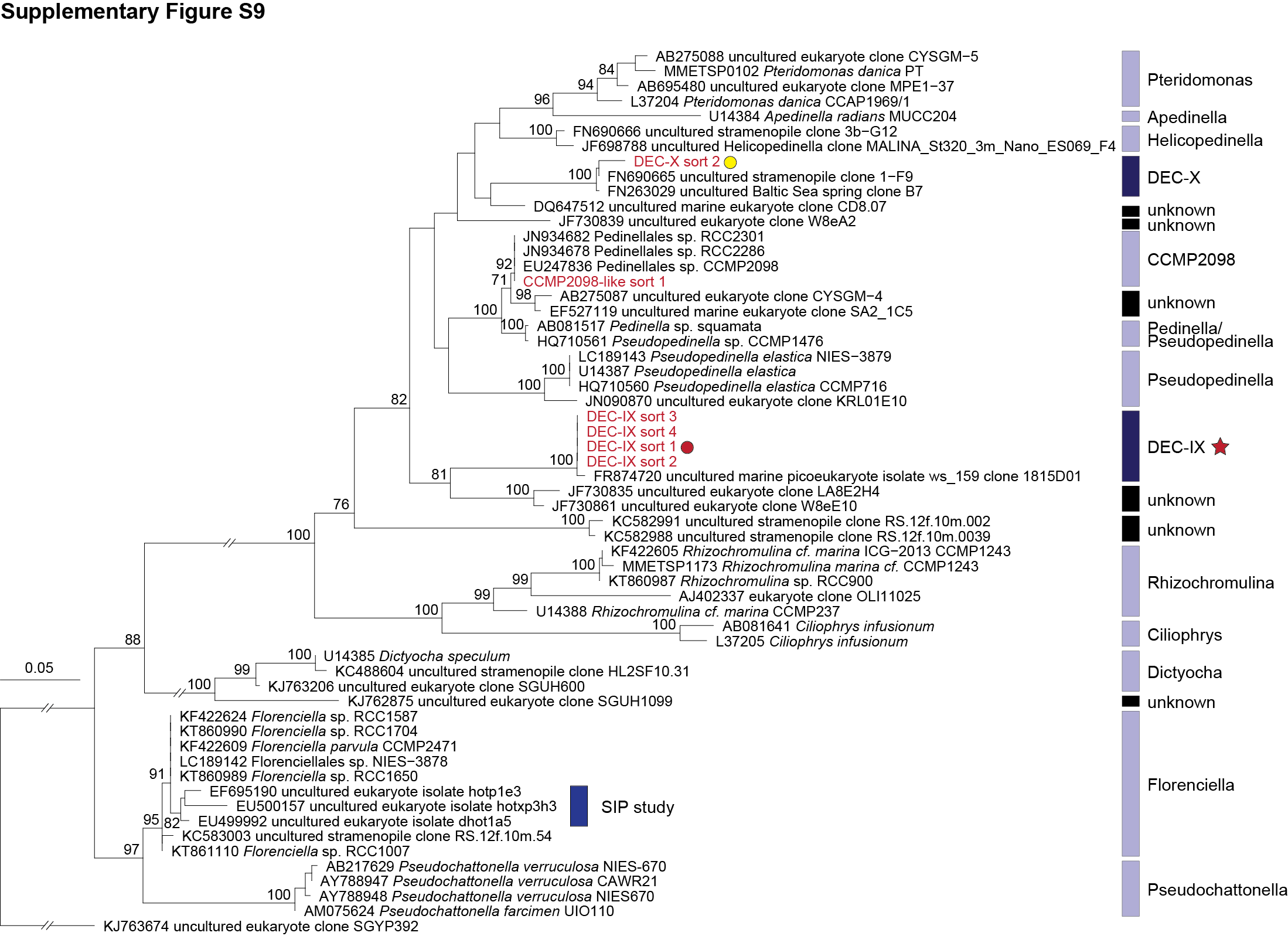


**Supplementary Figure S9** Phylogeny of dictyochophytes based on near full length 18S rRNA genes for all sequences except those from sorts in which the amplicon lengths were between 391 and 393 nt. Reference sequences representing *Florenciella* from a prior SIP study (Frias-Lopez et al., 2009) were included and indicated. The two sorted cells with a star (red) were sequenced to full length (1,788 nt). Colored circles represent the index sorting from Supplementary Figure S7A. Maximum Likelihood tree inferred by RAxML under gamma corrected GTR model of evolution was constructed based on 1,569 positions, and for amplicons use of missing positions (potentially resulting in biased placement). Node supports are nonparametric bootstrap percentages obtained from two different Maximum Likelihood inferences, 1,000 replicates from RAxML and 100 replicates from PhyML. Additional node statistical support was indicated based on the Bayesian posterior probabilities.


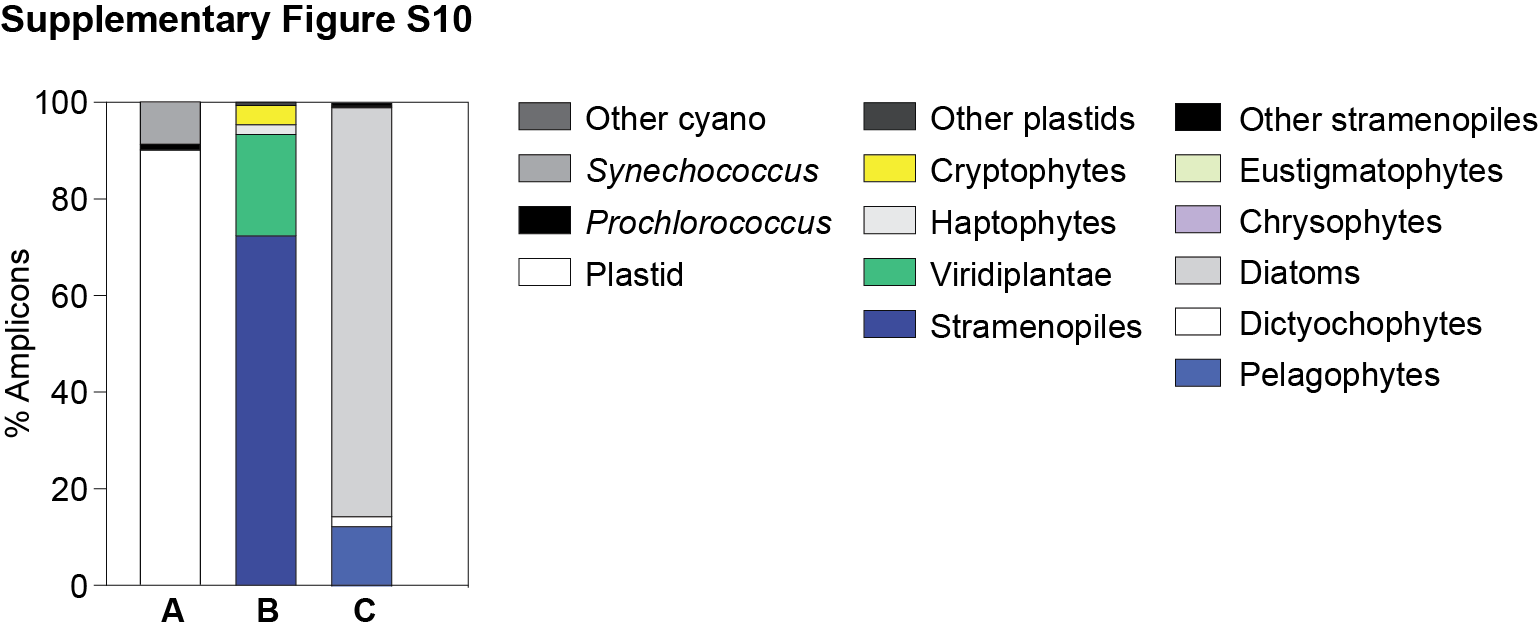


**Supplementary Figure S10** Phytoplankton distribution in coastal surface waters on the ENP Line-67. The relative abundance of overall phytoplankton 16S V1-V2 amplicons expressed as a percentage of amplicons phylogenetically assigned to **(A)** plastids, *Prochlorococcus*, *Synechococcus* and other cyanobacterial groups, **(B)** major eukaryotic phytoplankton lineages and **(C)** photosynthetic stramenopile classes.


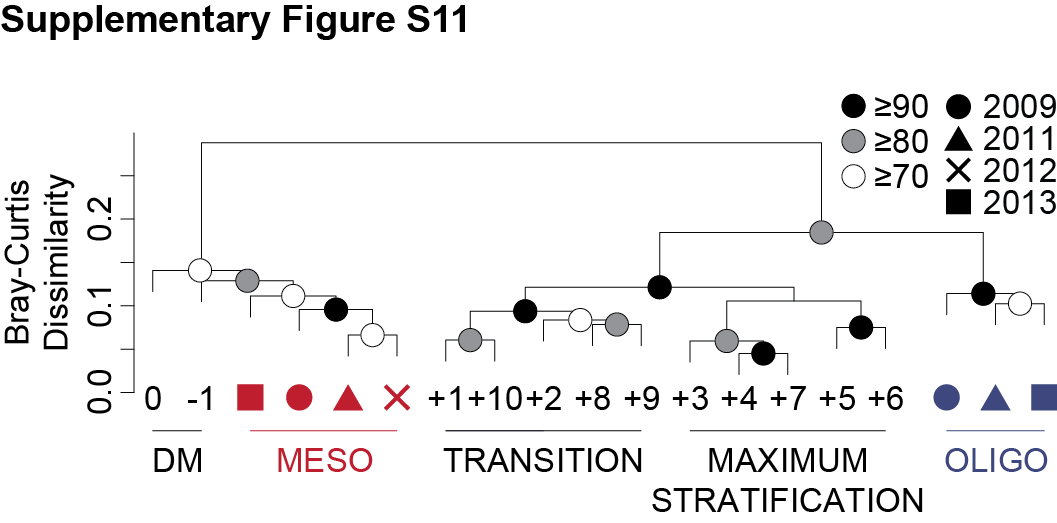


**Supplementary Figure S11** Hierarchical clustering analysis with Bray-Curtis dissimilarity based on the relative abundance of stramenopile amplicons in surface samples from BATS (averaged monthly data from month -1 to +10) and the ENP (red, mesotrophic stations; blue, oligotrophic stations). Approximately unbiased (AU) probability values based on the multiscale bootstrap resampling (10,000 replications) were calculated and expressed as *p*-values (%).

**
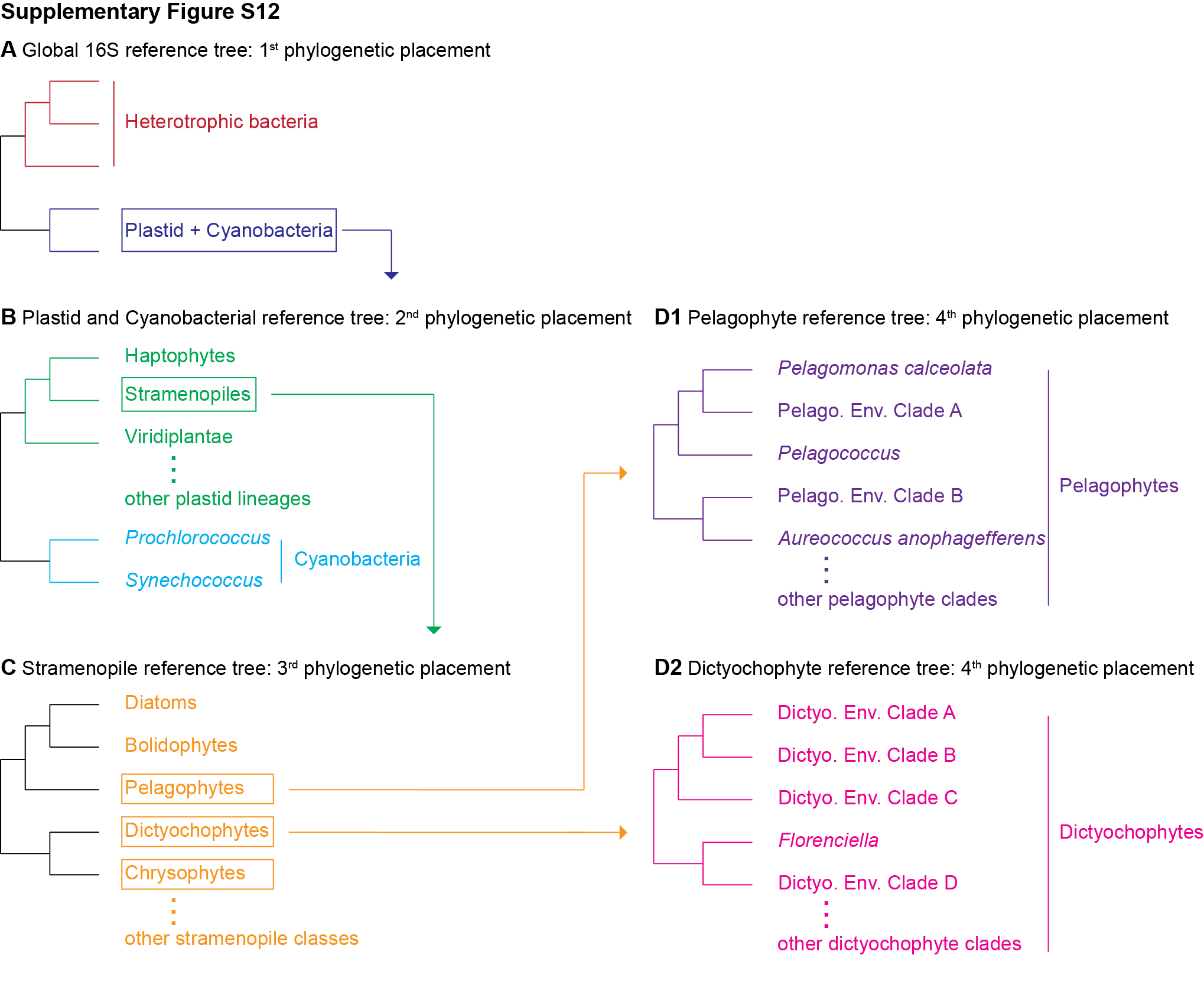
**

**Supplementary Figure S12** Overall description of 16S rRNA gene V1-V2 amplicon analysis for each phylogenetic groups and subgroups. **(A)** Phytoplankton 16S amplicons were classified based on the overall 16S reference tree. **(B)** Amplicons representing either plastids or cyanobacteria were binned with a global cyanobacterial and plastid reference tree. **(C)** Amplicons placed in the stramenopile cluster of the global cyanobacterial and plastid tree were retrieved and analyzed using the stramenopile reference tree. **(D)** Pelagophyte and dictyochophyte amplicons were further analyzed using pelagophyte and dictyochophyte reference trees.

**Supplementary Tables**

**Supplementary Table S1** The relative abundance of overall phytoplankton 16S V1-V2 amplicons expressed as a percentage of amplicons phylogenetically assigned to cyanobacteria and plastids at BATS.


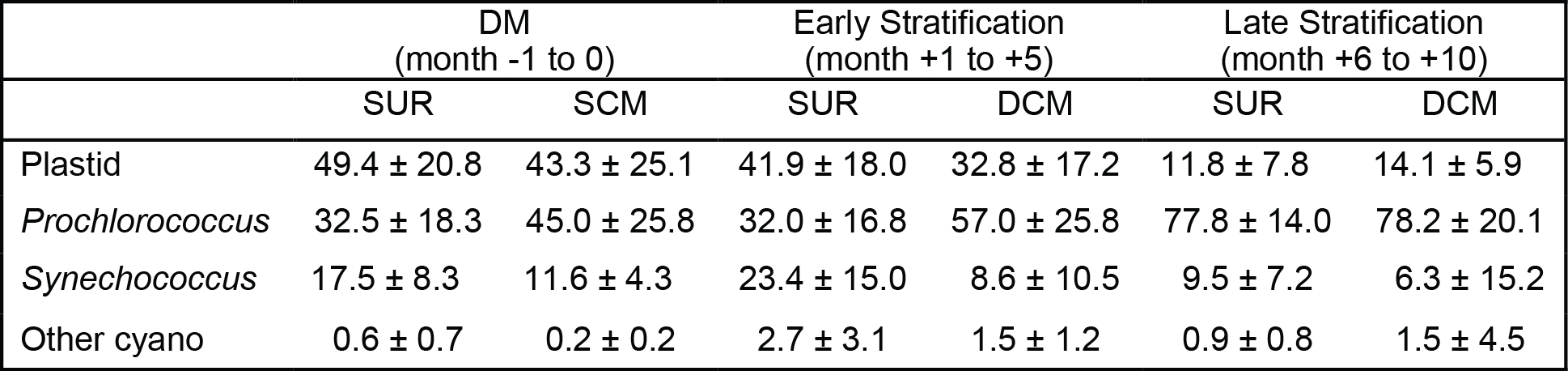


**Supplementary Table S2** The relative abundance of 16S V1-V2 amplicons expressed as a percentage of amplicons phylogenetically assigned to major photosynthetic eukaryotes at BATS.


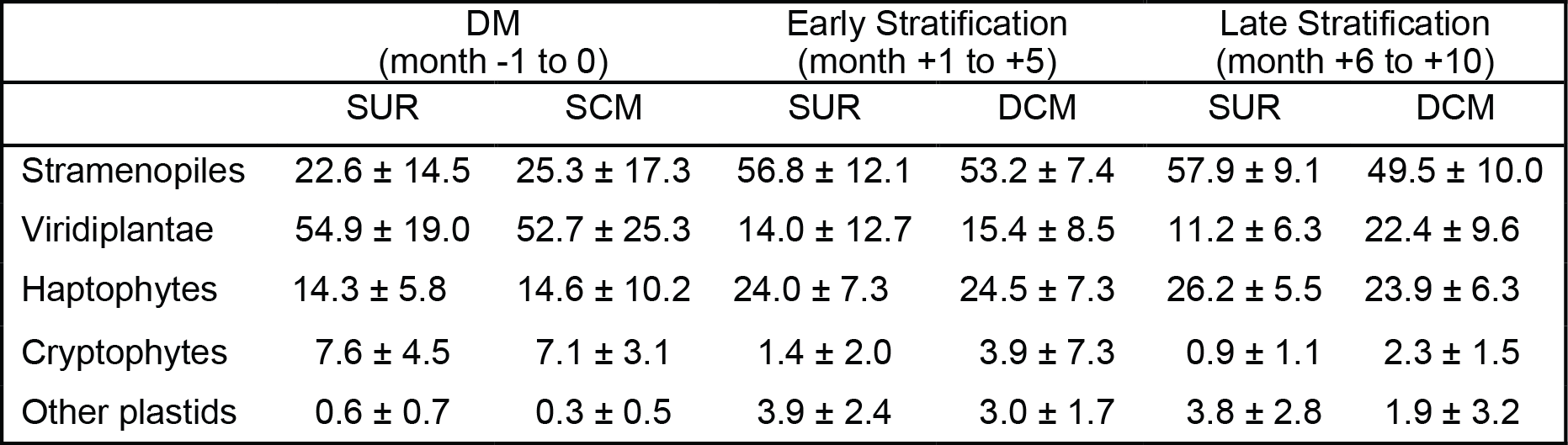


**Supplementary Table S3** The relative abundance of 16S V1-V2 amplicons expressed as a percentage of amplicons phylogenetically assigned to major stramenopile classes at BATS.


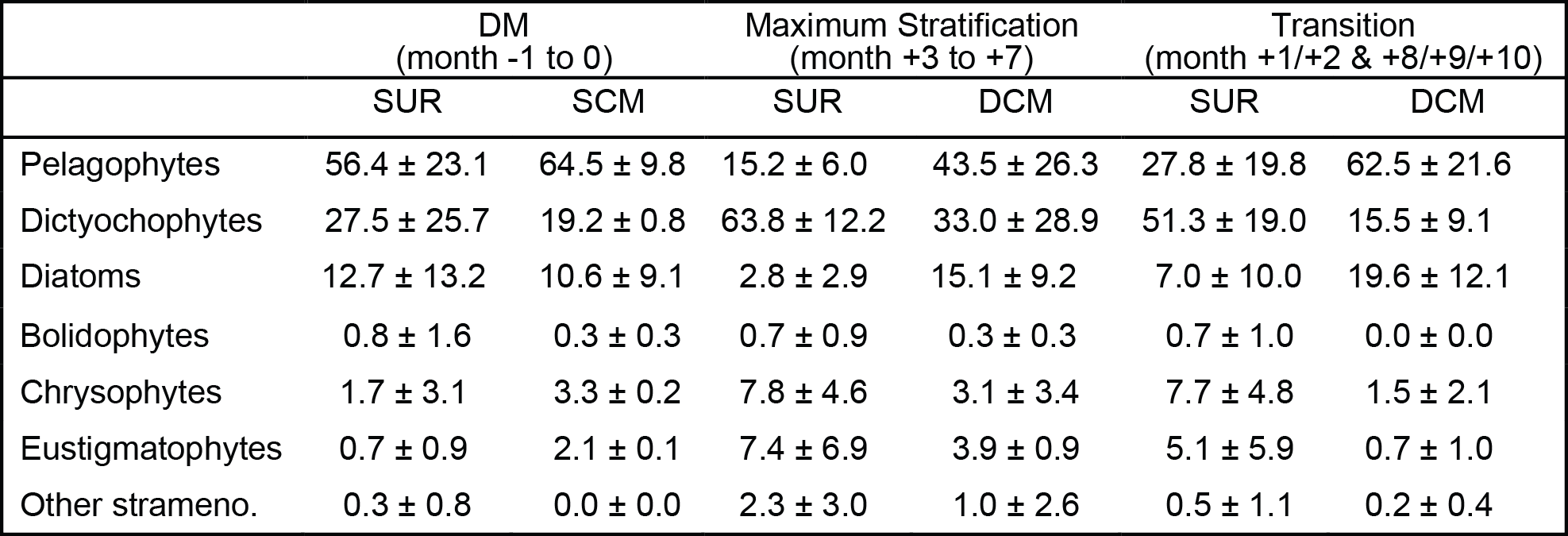


**Supplementary Table S4** Pelagophyte clade distribution in relation to depth during the months of deep mixing (DM) and Maximum Stratified periods at BATS. Two months are chosen as representatives for DM (month -1 and 0) and Maximum Stratified (month +4 and +6) periods.


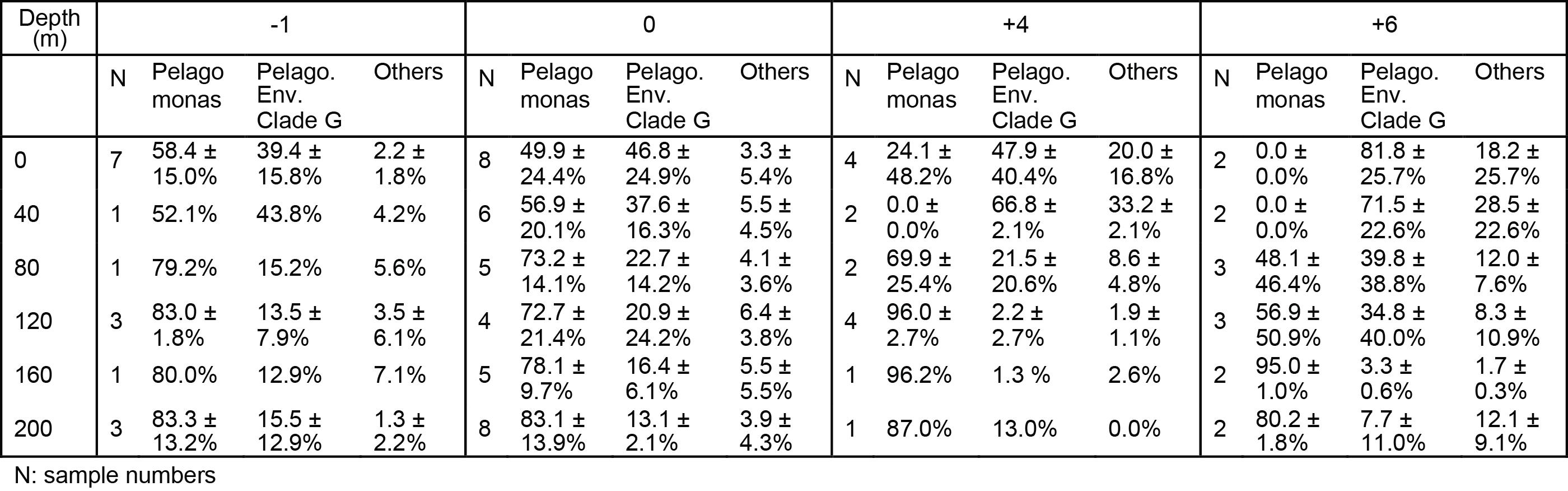


**Supplementary Table S5** Physico-chemical parameters attained for the analyzed amplicon sequencing samples. Note that some values have been published in (Sudek et al., 2015).


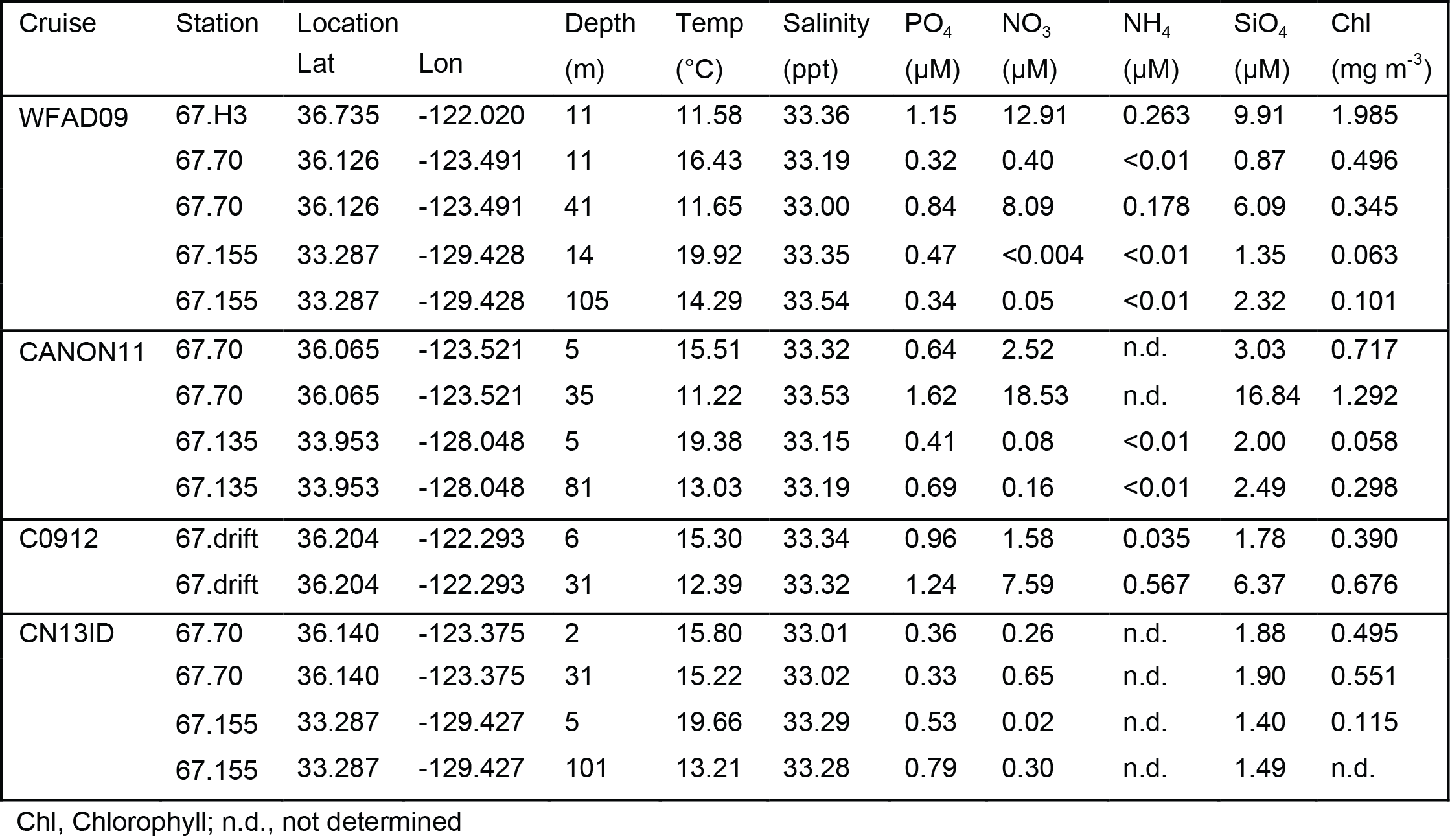


**Supplementary Table S6** *Pelagomonas calceolata* specific primer-probe set utilized for quantitative PCR analysis.


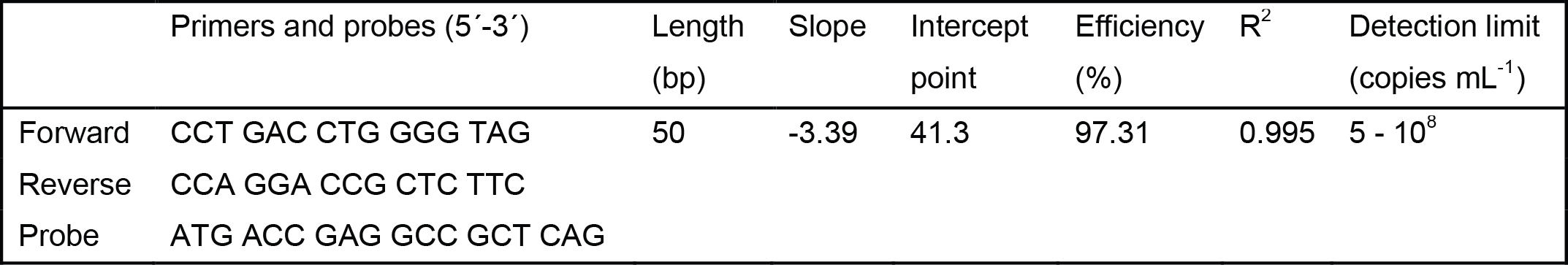


**Supplementary Table S7** Specificity validation of *P. calceolata* primer-probe set utilized for quantitative PCR analysis.


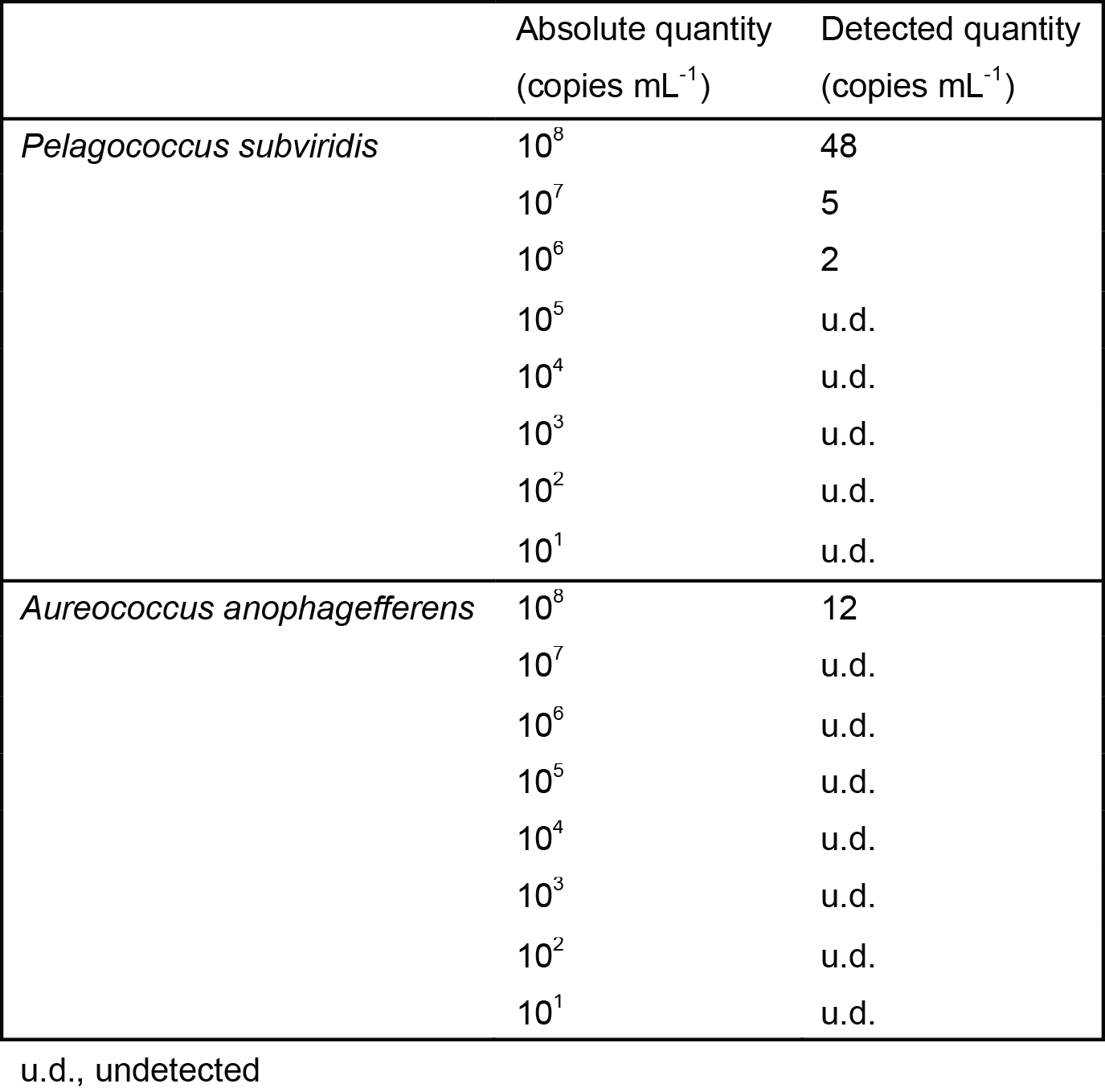


**Supplementary Table S8** Accession numbers for key thiamin biosynthesis genes found in the dictyochophyte transcriptomes available through MMETSP. Candidate genes were identified by TBLASTN (cut-off e^-5^ and minimum length 200 bp) using diatom *Thalassiosira pseudonana* and *Phaeodactylum tricornutum* as well as *Arabidopsis thaliana* genes as queries (also included was thiC from *Aureococcus anophagefferens* which does not have nmt1 or thiE). For confirmation, candidates were used as queries in BLASTX searches of GenBank nr database. Candidates that only generated bacterial hits or did not have relevant BLASTX hits with e^-15^ or lower expectation value were discounted.


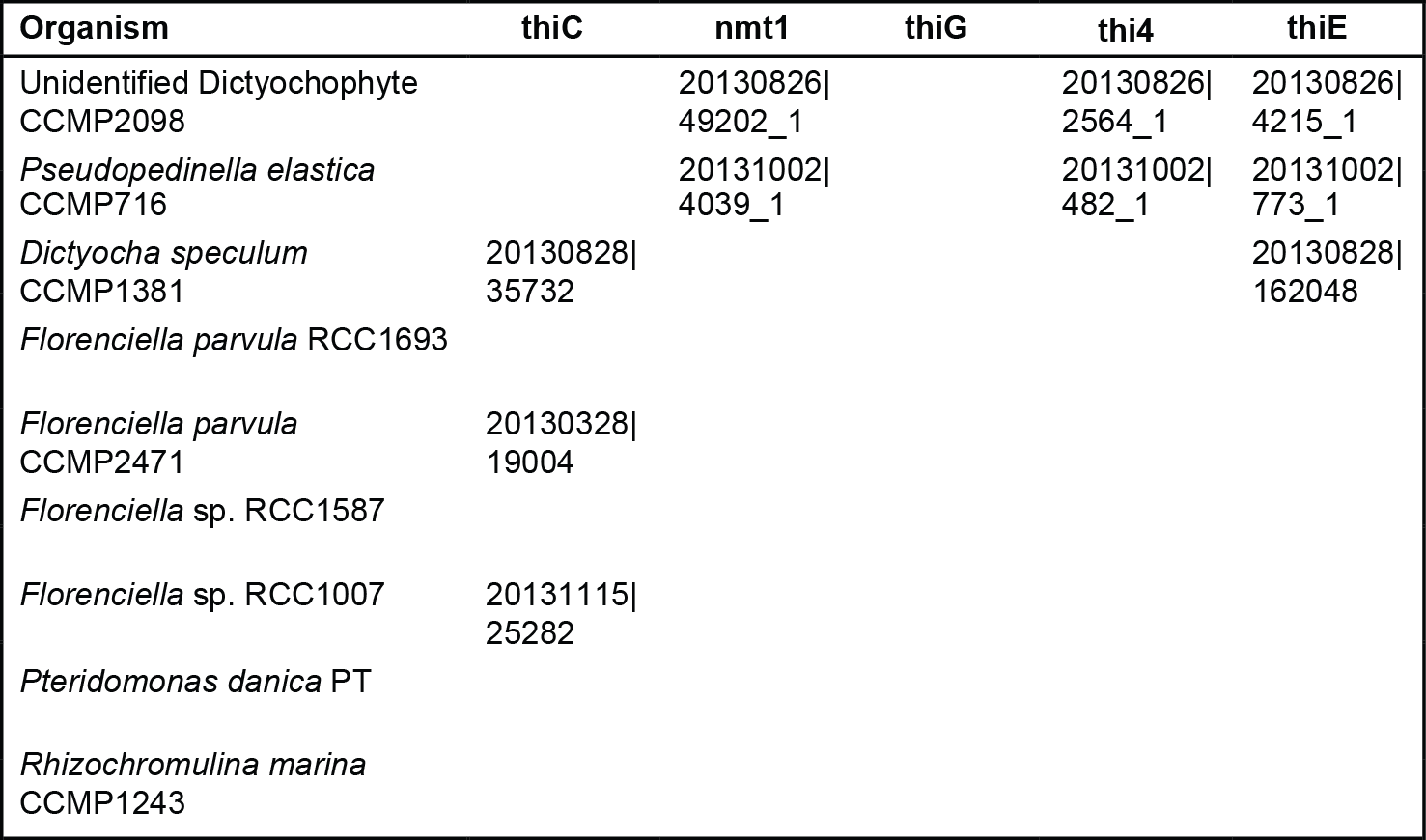


**Supplementary Table S9** Accessions and sample details.


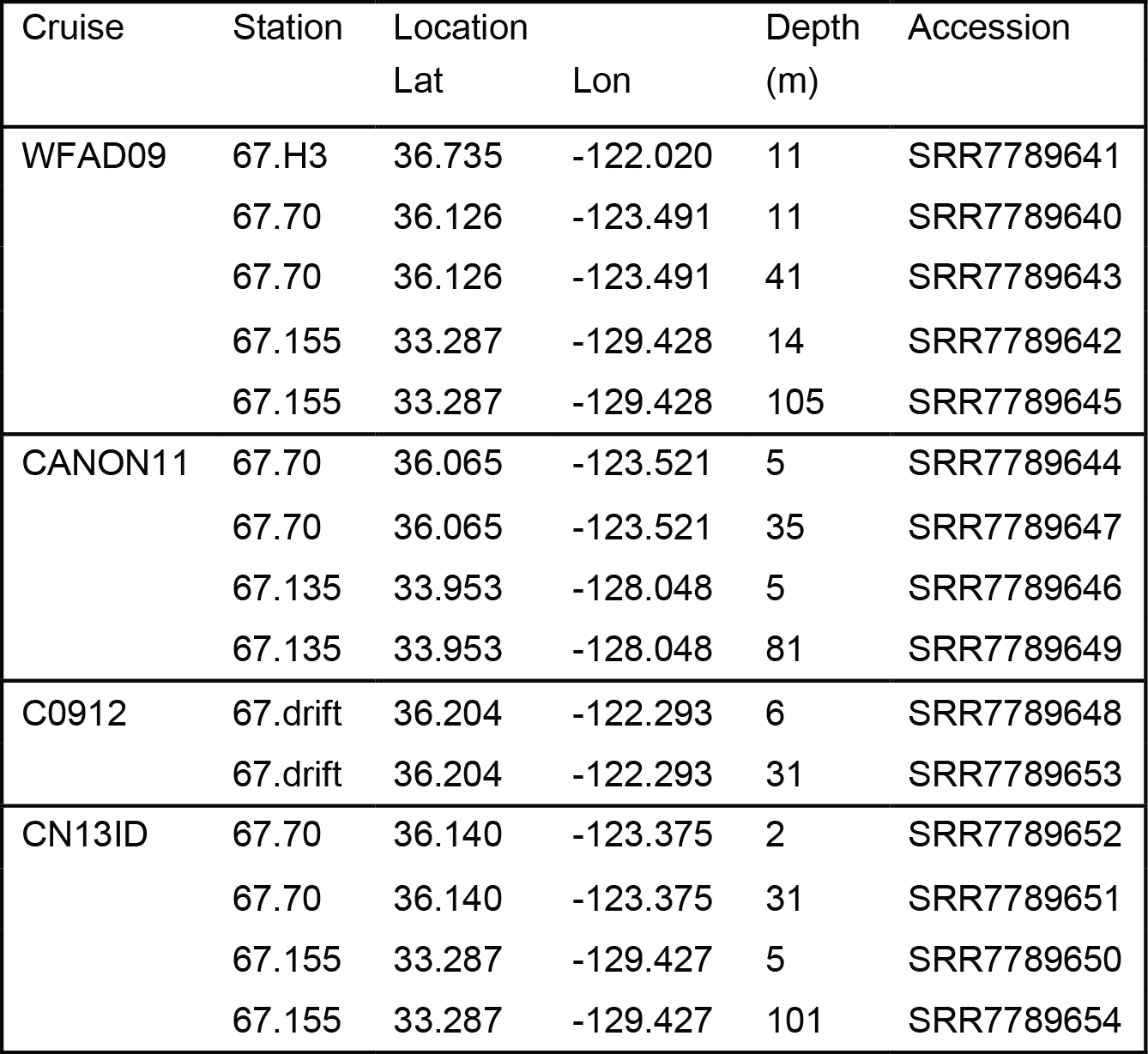

Supplement: Supplementary file 1 [file Data_Sheet_1.docx]
